# From Physiology to Pathology of Cortico-Thalamo-Cortical Oscillations: Astroglia as a Target for Further Research

**DOI:** 10.3389/fneur.2021.661408

**Published:** 2021-06-09

**Authors:** Davide Gobbo, Anja Scheller, Frank Kirchhoff

**Affiliations:** Molecular Physiology, Center for Integrative Physiology and Molecular Medicine (CIPMM), University of Saarland, Homburg, Germany

**Keywords:** astrocytes, sleep/wake cycle, NREM, network plasticity, cortico-thalamo-cortical oscillations, spike and wave discharges, sleep

## Abstract

The electrographic hallmark of childhood absence epilepsy (CAE) and other idiopathic forms of epilepsy are 2.5–4 Hz spike and wave discharges (SWDs) originating from abnormal electrical oscillations of the cortico-thalamo-cortical network. SWDs are generally associated with sudden and brief non-convulsive epileptic events mostly generating impairment of consciousness and correlating with attention and learning as well as cognitive deficits. To date, SWDs are known to arise from locally restricted imbalances of excitation and inhibition in the deep layers of the primary somatosensory cortex. SWDs propagate to the mostly GABAergic nucleus reticularis thalami (NRT) and the somatosensory thalamic nuclei that project back to the cortex, leading to the typical generalized spike and wave oscillations. Given their shared anatomical basis, SWDs have been originally considered the pathological transition of 11–16 Hz bursts of neural oscillatory activity (the so-called sleep spindles) occurring during Non-Rapid Eye Movement (NREM) sleep, but more recent research revealed fundamental functional differences between sleep spindles and SWDs, suggesting the latter could be more closely related to the slow (<1 Hz) oscillations alternating active (Up) and silent (Down) cortical activity and concomitantly occurring during NREM. Indeed, several lines of evidence support the fact that SWDs impair sleep architecture as well as sleep/wake cycles and sleep pressure, which, in turn, affect seizure circadian frequency and distribution. Given the accumulating evidence on the role of astroglia in the field of epilepsy in the modulation of excitation and inhibition in the brain as well as on the development of aberrant synchronous network activity, we aim at pointing at putative contributions of astrocytes to the physiology of slow-wave sleep and to the pathology of SWDs. Particularly, we will address the astroglial functions known to be involved in the control of network excitability and synchronicity and so far mainly addressed in the context of convulsive seizures, namely (i) interstitial fluid homeostasis, (ii) K^+^ clearance and neurotransmitter uptake from the extracellular space and the synaptic cleft, (iii) gap junction mechanical and functional coupling as well as hemichannel function, (iv) gliotransmission, (v) astroglial Ca^2+^ signaling and downstream effectors, (vi) reactive astrogliosis and cytokine release.

## Introduction

Epilepsy is a highly heterogeneous neurological condition characterized by enduring predisposition to unpredictable pathological discharge of rhythmic activity in the brain networks, which is commonly referred as seizure activity (1). In virtue of the severity and nature of the pathological alteration (abnormal, excessive, or excessively synchronous activation) as well as the cellular and anatomical composition of the affected brain networks, seizures can cause changes in the level of consciousness, behavior, memory, and emotional status. Although the etiology of epileptiform activity is still unknown in half of the cases, understanding the pathological alteration at the basis of the epileptic phenotype may not only be of fundamental therapeutical importance but also provide further insights into the functioning of the affected neural networks in the physiology of the healthy brain. The identification of the molecular and cellular mechanisms underlying physiological oscillations is critical for a full comprehension of their relationship to the respective pathological activity. In this regard, an exceptional case of study is the cortico-thalamo-cortical network, physiologically engaged during sleep and pathologically altered in the context of non-motor (absence) seizures (2).

Absence seizures are transient non-convulsive generalized epileptic events and are also referred as *petit mal* seizures (2, 3). Phenotypically, absence seizures are coupled with sudden and brief impairment of consciousness and lack of responsiveness to external stimuli as well as variable secondary clinical symptoms (e.g., automatisms, atonic, and tonic muscular components etc.) (4, 5). Absence seizures are the sole clinical symptom of childhood absence epilepsy (CAE) but are also associated with several other idiopathic generalized epilepsies (4, 6–11). Although CAE has up to 70% remission rate (7, 12), the gold standard monotherapy, based on ethosuximide and valproic acid, is still ineffective in 30% of the cases (13). Moreover, clinical conditions displaying absence seizures are often associated with severe neuropsychiatric comorbid conditions such as impaired attention, learning, memory and cognition, which are often left unaltered or even worsened by common antiepileptic drugs (14–17).

**Figure 1:**
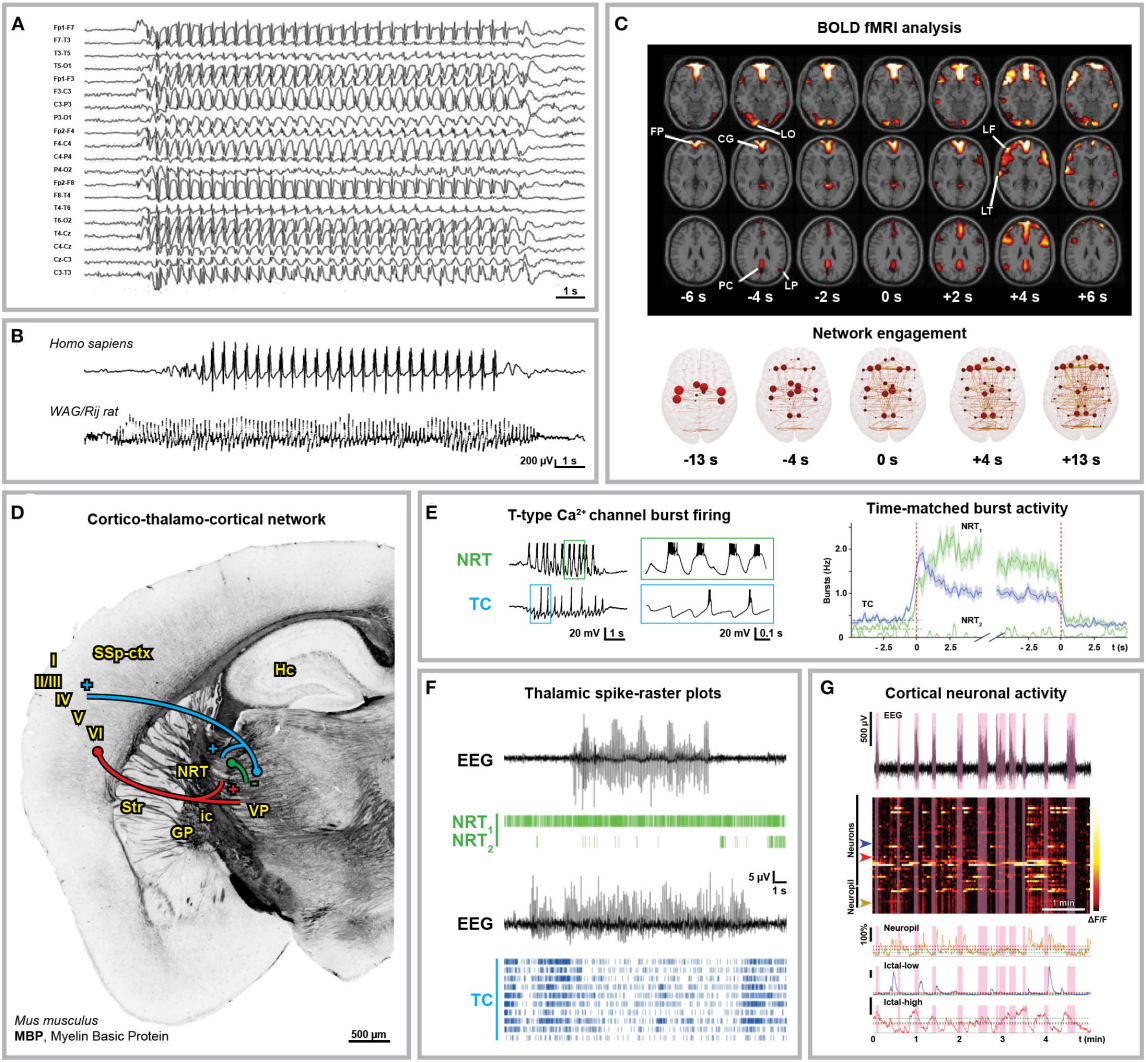
Anatomical and electrophysiological characterization of absence seizures. **(A)** Human electroencephalographical recording (EEG) displaying typical 3 Hz spike-and-wave discharges (SWDs). **(B)** 3 Hz SWDs associated with Childhood Absence Epilepsy (top trace, 8-year-old boy) and 8 Hz SWDs recorded in an adult WAG/Rij rat (bottom trace). **(C)** Blood Oxygenation Level Dependent (BOLD) functional Magnetic Resonance Imaging (fMRI) changes associated with absence seizures before (FP, frontal polar; CG, cingulate; LO, lateral occipital; PC, precuneus; LP, lateral parietal cortex) and after (LF, lateral frontal; LT, lateral temporal cortex) seizure onset (top) and brain network engagement analysis around SWDs events (bottom). **(D)** Anatomical organization of the cortico-thalamo-cortical network in C57BL/6N mice. Myelin Basic Protein (MBP) immunostaining indicates the axonal fiber tracts connecting the network (own data; mouse monoclonal MBP antibody, BioLegend, AB_2564741, Cat. No. 808401, 1:500). Cortico-thalamic excitatory neurons from cortical layers V and VI (red) project to both NRT and thalamus; thalamo-cortical excitatory neurons (blue) project back to cortical layer IV and NRT; NRT GABAergic neurons (green) inhibit the thalamic nuclei. I-VI, cortical layers; GP, globus pallidus; Hc, hippocampus; ic, internal capsule; NRT, nucleus reticularis thalami; SSp-ctx, primary somatosensory cortex; Str, striatum; VP, ventral posterior thalamic nuclei. **(E)** Glutamatergic thalamo-cortical neurons (TC) as well as GABAergic NRT neurons display T-type Ca^2+^ channel-mediated burst firing during SWDs (left, *ex vivo* recording from ferret thalamic slices). **(F)** Spike-time raster plots of two representative NRT neurons (NRT_1_, top trace; NRT_2_, bottom trace) and 10 TC neurons with time-matched EEG in GAERS rats. The overall TC activity decreases during SWDs and only a small portion of TC neurons fire synchronously. **(G)** 2-Photon laser scanning microscopy of neuronal cortical Ca^2+^ activity in *stargazer* mice during absence seizures. Heatmap of neuronal Ca^2+^ activity shows that only a subpopulation of neurons displays ictal synchronous firing. Modified from **(A)**, (19); **(B)**, (20, 21); **(C)**, (22, 23); **(E)**, (24, 25); **(F)**, (25); **(G)**, (26).

Although absence seizures display inter- and intraindividual variability (17, 18), they exhibit generalized bilateral 2.5–4 Hz spike and wave discharges (SWDs) with no aura or post-ictal depression (Figures 1A,B) (4, 27–29). It is widely accepted that the sharp spike and the slow wave component of SWDs are functionally coupled and correspond to a state of neuronal excitation and silence in the cortico-thalamo-cortical network, respectively (30). Blood oxygenation level-dependent (BOLD) functional magnetic resonance imaging (fMRI) studies in humans consistently showed cortical network engagement in correspondence of and even preceding the appearance of SWDs in electrographic traces as well as an increased interictal synchrony in the sensorimotor cortex (Figure 1C) (22, 23, 31–36). Most advancements on the understanding of the cellular and synaptic mechanisms underlying SWDs derive from the extensive use of genetic animal models, particularly the genetic absence epilepsy rats from Strasbourg (GAERS) and Wistar-Albino-Glaxo rats from Rijswijk (WAG/Rij) (20, 37–42) as well as many monogenic mouse mutants (43–45). Although sharing most electrographic and behavioral hallmarks of absence seizures, animal models are characterized by higher SWD frequencies (5–11 Hz) (Figure 1B). E*x vivo* multi-site local field potential studies identified the peri-oral primary somatosensory cortex as initiation site of absence seizures in WAG/Rij (46) and GAERS rats (47–50). Notably, this has been proven wrong for the acute pharmacological γ-hydroxybutyric acid (GHB) model (51–54) in mice, where the prefrontal cortex was suggested as the initiation site of SWDs (55). With this in mind and considering the many areas contributing to the cortical pre-ictal BOLD changes of absence seizures, one can probably not identify a unique canonical focal onset or initiation site for absence seizures. Instead, the denomination *cortical initiation network* has been recently proposed (17), thereby settling the long-standing controversy about the SWDs initiation site (56–59). However, the existence of a cortical initiation network does not imply that manipulation of the sole thalamic components of the cortico-thalamo-cortical network is not sufficient to induce SWDs, as it is indeed the case (60–62), or that the wide thalamo-cortical innervation is not crucial for SWDs generalization, as suggested by the existence of subclinical SWDs restricted to the cortical network (48). In particular, the thalamic posterior nucleus plays a crucial role in the generalization of SWDs (61, 63–66). Till recently, *ex vivo* studies performed in different mammalian models identified the hyperexcitability and T-type Ca^2+^ channel-mediated burst activity of glutamatergic thalamo-cortical neurons and GABAergic neurons from the thalamic reticular nucleus (or *nucleus reticularis thalami*, NRT) as the rhythmogenic cortico-thalamo-cortical network mechanism of SWDs (Figures 1D,E) (24, 41, 67–71). Nevertheless, recent *in vivo* studies performed in rodents showed that only a small fraction of thalamo-cortical and cortico-thalamic neurons are synchronously active at each SWD cycle and the cellular composition of this neuronal subpopulation changes between subsequent cycles, thus excluding the existence of distinctive neuronal subpopulations (Figures 1F,G) (25, 26). This explains why, with SWD progression, the activities of the cortico-thalamic and thalamo-cortical neurons undergo a phase-shift in time (46) since different neuronal subpopulations participate in this excitatory feedback-loop with slightly different kinetics. Moreover, this progressive phase-shift between different subpopulations active at the same time accounts for the overlapping average electrical activity in the cortico-thalamic, thalamo-cortical, and NRT neurons within any SWD cycle. Moreover, although interictal T-type Ca^2+^ channel burst activity in the thalamo-cortical neurons increases right before SWD onset, overall *in vivo* ictal thalamic activity decreases and only cortical and NRT T-type channels are essentials for SWDs (25). Interestingly, all NRT neurons fire within each SWD cycle, even though a fraction of those neurons fires relatively asynchronous tonic spikes rather than T-type Ca^2+^ channel-mediated bursts in phase with the SWDs (Figures 1E,F) (25). The enhanced tonic inhibition of thalamo-cortical neurons as well as the increased thalamic GABA level are key aspects of absence seizures (25, 72–78). Moreover, the fact that SWDs can be induced by the impairment of the cortico-thalamic glutamate release due to deletion of P/Q-type Ca^2+^ channels in the projecting cortical neurons from layer VI could suggest that a balance shift toward GABAergic inhibition more than an absolute increase of GABA levels is the key mechanism of SWD generalization (79). Additionally, the decreased glutamate release could lead to reduced activity of cortical interneurons, thus contributing to cortical hyperexcitability.

## Astrocytes Contribute to Network Priming and Synchronization as Well as Swd Induction, Propagation, and Termination

After more than three decades of accumulating evidence, nowadays it is widely established that astroglia constitute a ubiquitous non-neuronal communication system in the brain involved in virtually every physiological and pathological scenario of the central nervous system (80–82). Not only do they support synapses from a mechanical, metabolical as well as functional point of view, but they also participate in synaptic transmission and plasticity, neural network excitability and balance between excitation and inhibition (E/I) as active information integrators and processors (83–86). The contribution of the astroglial network to the pathophysiology of epilepsy encompasses a plethora of different molecular mechanisms which currently represent one of the most fruitful research topics in neuroscience (87–96). Pathological priming mechanisms of the astroglial network ultimately involve either E/I imbalance or enhanced network synchronization (or both simultaneously). Alternatively, astrocytes may influence spatial and temporal propagation of seizures, thus playing a key role in the phenotypical outcome of seizures and their severity and therefore representing a promising target for the development of new non-neurocentric drugs. Most of the recent evidence focuses on astroglial contribution to convulsive epileptic activity. Nevertheless, we discuss in the following the putative involvement of astrocytes in network priming as well as seizure induction and propagation which could have a role in pathological epileptic scenarios including SWDs, as well. We focus on the evidence that links to observations coming from the clinics as well as genetic and pharmacological models of SWDs, aiming to point at specific topics which may be worth further research in the field of SWDs.

### The Astroglial Network Controls Extracellular Space Homeostasis Through K^+^, Water and Solute Clearance

**Figure 2:**
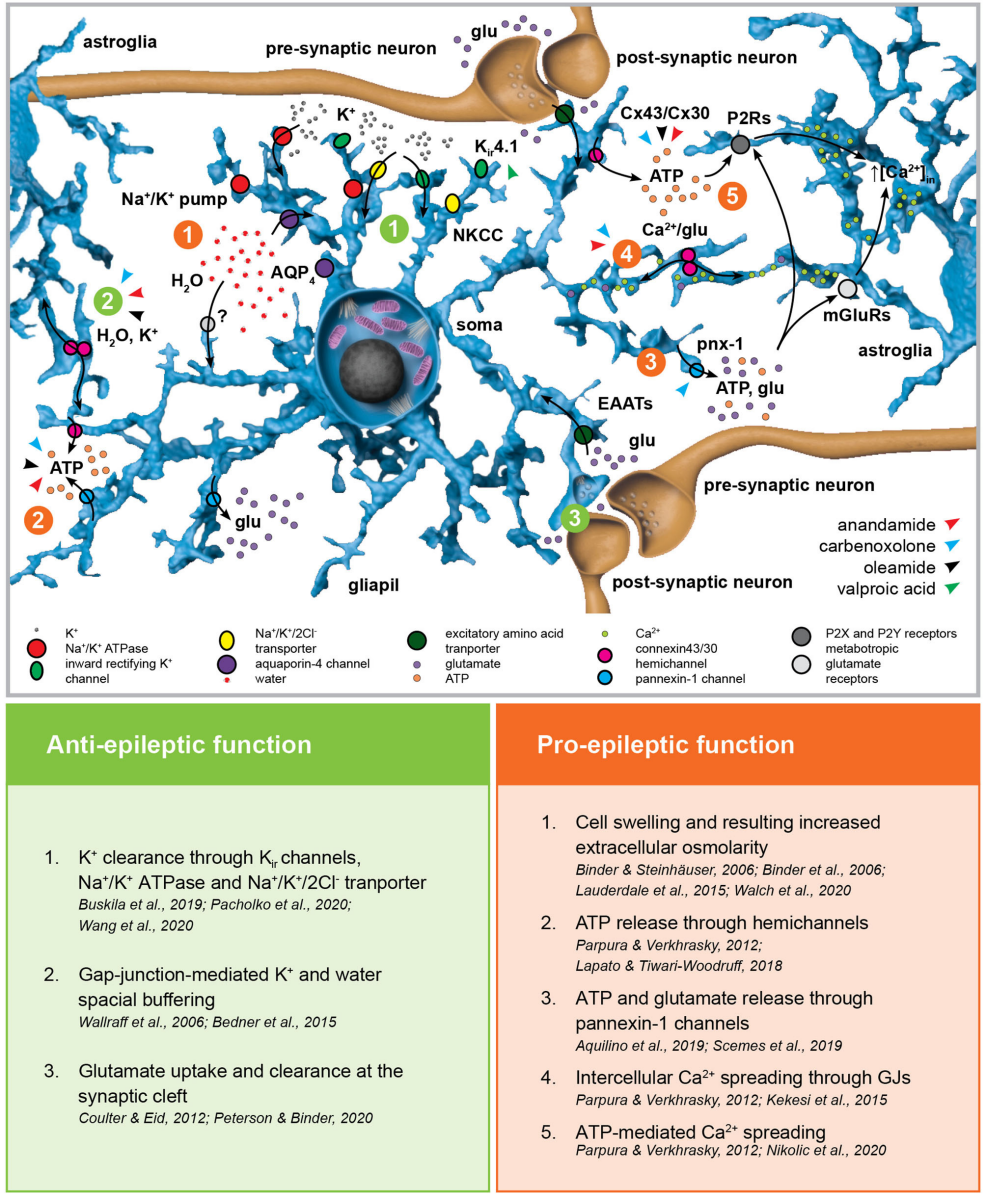
Astroglial homeostatic control of the extracellular space has opposite effects on epileptogenesis. The astroglial network is responsible for extracellular K^+^ uptake by means of inward rectifying K^+^ channels (K_ir_), Na^+^/K^+^ pump and Na^+^/K^+^/2Cl^−^ transporter (NKCC). K^+^ clearance is coupled with water uptake through the water channel aquaporin-4 (AQP4) and possibly *via* yet unknown additional pathways. The excitatory amino acid transporters EAAT1 and EAAT2 are responsible for glutamate uptake. Astroglial connexins Cx43 and Cx30 enable gap-junction (GJ) coupling responsible for spatial ionic and metabolic buffering. Connexin hemichannels as well as pannexin-1 channels (Panx1) mediate glutamate and ATP release in the extracellular space possibly activating astroglial metabotropic glutamate receptors (mGluRs) and purinergic P2X and P2Y receptors (P2Rs), respectively. This, in term, induces intracellular Ca^2+^ increases in the neighboring astroglia. The figure summarizes the pro- and anti-epileptic roles of the mechanisms described above and points to the putative targets of valproic acid and the GJ blockers carbenoxolone, anandamide, and oleamide in this scenario.

By means of their close juxtaposition to synapses, their expression of an extraordinary assortment of membrane transporters and receptors as well as their physical and functional coupling through gap junctions (GJs), astroglial networks provide a perfect spatial buffering for neural activity (Figure 2) (97–99). Astrocytes are key regulators of the extracellular K^+^ concentration. Their high K^+^ permeability mediated by inwardly-rectifying K_ir_ and two-pore-domain K_2P_ channels, Na^+^/K^+^ pumps and Na^+^/K^+^/Cl^−^ transporters associated with their extensive GJ coupling enables them to uptake and redistribute excessive extracellular K^+^ resulting from neuronal firing (100–103). Astroglial K^+^ and glutamate uptake is altered in cultured cortical astrocytes after K_ir_4.1 channel downregulation (104) as well as in astroglial-specific K_ir_4.1 knock-out mice (105, 106). Gain-of-function (107) as well as loss-of-function mutations (108, 109) in the human *KCNJ10* gene encoding K_ir_4.1 have been linked to forms of childhood epilepsies associated with ataxia and cognitive impairment, but not to CAE. Notably, artificially increasing extracellular K^+^ concentration *ex vivo* is associated with propagating epileptiform discharges induced by focal optogenetic activation of parvalbumin-expressing interneurons (110). *In vivo* though, K^+^ clearance impairment induced by blocking GJ was not sufficient to induce neocortical seizures (111). Interestingly, valproic acid (but not ethosuximide) induces K_ir_4.1 overexpression in the cortex of healthy rats (112). Nevertheless, further research is required to address the actual contribution of K_ir_4.1 overexpression in the anti-absence effects of valproic acid, as well as the putative role of astroglial K_ir_4.1 itself in the development and propagation of SWDs. The combined use of cell-specific conditional knock-out of these channels and pharmacological models of SWDs could shine new light on the topic.

K^+^ uptake is associated with cellular swelling due to Na^+^/K^+^-pump dependent water influx (113). Although the exact molecular mechanisms are still under debate, water fluxes across the astrocytic membrane are associated with K^+^ homeostasis and they influence local interstitial osmolarity as well as seizure generation and progression (114–120). Given the accumulating evidence against the predominant contribution of the astroglial water channel aquaporin-4 (AQP4) in water homeostasis (113, 121), the use of AQP4 conditional knock-out as a model of disrupted water homeostasis has been recently challenged. Nevertheless, water homeostasis impairment and the resulting volume and osmolarity dysregulation should affect neural network excitability. Indeed, a recent structural MRI study on CAE showed significant gray matter volume abnormalities in both frontotemporal cortical region and posterior thalami compared to controls (122, 123).

GJ coupling, particularly mediated by connexins Cx30 and Cx43, provides the astroglial network with a high level of intercellular structural, metabolic and functional connectivity, enabling the exchange of ions and small molecules (124–131). In the context of epilepsy, connexins mediate ATP release (into the extracellular space through hemichannels), the spreading of intercellular Ca^2+^ waves (132) and are fundamental in the spatial buffering required for K^+^ and water homeostasis as well as glutamate clearance (133, 134). With respect to absence epilepsy, most advancement in unraveling the role of GJs has been obtained employing GJ blockers in well-established genetic animal models (135). The broad-spectrum GJ blocker carbenoxolone (CBX) decreased both amplitude and duration of 4-aminopyridine-induced seizure-like events (SLEs) in thalamocortical slices obtained from mice with spontaneous SWDs (136, 137) as well as the duration of SWDs seen in GAERS rats *in vivo* after systemic application (138). Interestingly, *in vivo* injection of CBX in the NRT of rats with atypical absence seizures and spontaneous SWDs decreased the duration of SWDs (139), whereas no alteration of SWD phenotype was observed if CBX was injected in the posterior thalami of WAG/Rij rats and the *lethargic* mouse genetic model of absence epilepsy (140). Recently, intraperitoneal injection of CBX was associated with absence seizures worsening in WAG/Rij rats (141), hinting at non-obvious and non-trivial differences across the absence epilepsy models. The endocannabinoids anandamide (*N*-arachidonoylethanolamine, ANA) and oleamide (cis-9,10-octadecenoamide, OLE) are specific Cx43 blockers (142, 143). Intracerebroventricular injection of ANA decreased in a dose-dependent manner the recurrence and duration of SWDs, although its mechanism of action likely involves type-1 cannabinoid (CB1) receptor activation (144) or even direct inhibition of T-type Ca^2+^ channels (145). Interestingly, although specific studies addressing the impact of OLE in absence epilepsy are still required, OLE has a sleep-inducing effect and enhances GABA_A_ receptor-mediated responses, thus possibly affecting the physiological, temporal-spatial pattern of cortico-thalamo-cortical oscillations (146, 147). CBX as well as ANA and OLE block both GJ activity and connexin hemichannels regulating water and solute (notably ATP) exchanges between the intra- and extracellular space, thus challenging the attribution of any observed phenotype to the sole GJ coupling (131, 148). Moreover, regional differences in connexin isoform expression may be at the basis of different contributions of GJ and hemichannel inhibition in different neural networks, and thus the net phenotypical outcome of the pharmacological manipulation (149). CBX is also known to block pannexin-1, which bears significant topological and pharmacological similarities with the connexins and forms single-membrane channels which have been linked to network hyperexcitability and hypersynchronization by mediating both ATP and glutamate release (150, 151). The use of antibodies or small peptides targeting specific amino acid sequences of different connexins (152–155) could shed new light into the differential contribution of GJ coupling and hemichannel function as well as into the role of different connexin isoforms and pannexin-1 channels in the generation and propagation of SWDs. Finally, ANA, but not OLE, can block Ca^2+^ wave propagation in astrocytes, which has to be taken into consideration in the interpretation of the results (142, 143).

*In summary, astroglial networks contribute to the imbalance of neural excitation/inhibition through K*^+^ *and neurotransmitter (glutamate but also GABA) clearance under physiological conditions, thus counteracting network priming through aberrant shifts in the E/I balance possibly leading to network synchronization. Astrocytes rely on their extensive GJ coupling enabling effective spatial ionic, osmotic, and functional buffering. GJ hemichannels as well as pannexin-1 channels may be responsible for augmented synchronous activity through ATP and glutamate release and following Ca*^*2*^ *spreading throughout the astroglial network. So far, we are still missing evidence for linking the astroglial fine-tuning of the extracellular ion and transmitter homeostasis to SWDs. However, as it is the case for other kinds of epileptiform activity, their role in regulating such network excitability is very likely*.

### Astrocytes Are Actively Involved in Network Dynamics and E/I Balance Through Neurometabolic Coupling, Neurotransmission Modulation and Gliotransmission

**Figure 3:**
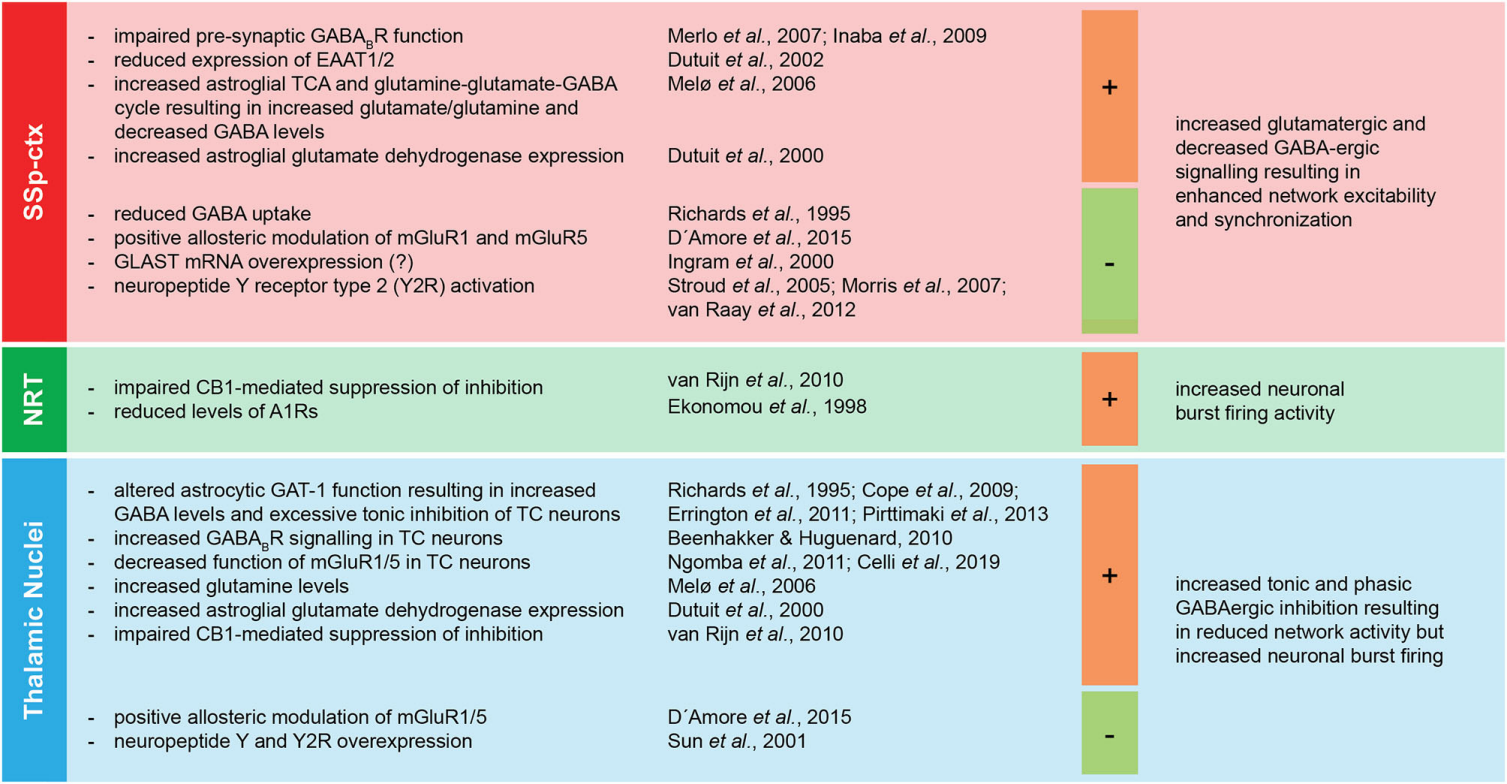
Regional specific imbalance of E/I at the basis of absence seizures. The main components of the cortico-thalamo-cortical network (SSp-ctx, primary somatosensory cortex; NRT, nucleus reticularis thalami; thalamic nuclei) display regional specific shifts toward either excitation or inhibition associated with absence seizures. The figure summarizes the pro- (+) and anti- (-) epileptic effects of specific alterations of the regional E/I balance in the pathological phenotype of absence seizures. A1R, adenosine receptor type 1; EAAT1/2, excitatory amino acid transporters 1/2; CB1, cannabinoid receptor type 1; GABA, γ-aminobutyric acid; GABA_B_R, metabotropic GABA_B_ receptor; GAT-1, GABA transporter 1; mGluR1/5, metabotropic glutamate receptors 1/5; TC, thalamo-cortical; TCA, tricarboxylic acid cycle; Y2R, neuropeptide Y receptor type 2.

Astrocytes do not only contribute to neural excitability and functioning by responding to neurotransmitter release and modification of extracellular ionic composition, they are also actively involved in neurotransmitter uptake and release, thus having a direct control of E/I balance (Figure 3) (86, 156–158). One of the key features of absence epilepsy are altered GABA levels (72, 159) and GABAergic tonic and phasic inhibition in the cortico-thalamo-cortical network (25, 73). In both GAERS rats and *stargazer* mice, astroglial GABA transporter GAT-1 malfunction leads to increased GABA levels in the thalamus resulting in altered tonic inhibition of GABA_A_ receptors on the thalamo-cortical neurons (72, 74, 75, 77). Notably, a number of human mutations in *SLC6A1* encoding GAT-1 leads to reduced GABA transport activity, and some of the mutations are associated with CAE or clinical conditions associated with absence seizures (160–164). Moreover, GABA released by astrocytes was proven to activate GABA_A_ receptors on the membrane of thalamocortical neurons in rodents (165) and blocking astroglial GATs increased extrasynaptic GABA_A_ receptor-mediated tonic inhibition (166). On the other hand, thalamic astrocytes express GABA_A_ receptors themselves (167), whose specific role has not been fully resolved yet. Neuronal presynaptic GABA_B_ receptor expression and function is impaired in the neocortex of WAG/Rij rats, possibly contributing to network hyperexcitability (168, 169). There is plenty of evidence that GABA_B_ receptors contribute to network priming in absence seizures facilitating thalamo-cortical burst firing, as supported by the exacerbation of SWDs after baclofen or GHB treatment (170–176). Interestingly, the activation of extrasynaptic GABA_B_ receptors require GABA spillover resulting from an intense GABAergic stimulation, which is in accordance with a predominant role of astrocytic GAT-1 in regulating SWDs, given its expression in close proximity of neuronal synapses compared to a more distal location of GAT-3 (175). A further level of complexity is given by the fact that astrocytes themselves express GABA_B_ receptors and their activation leads to downstream Ca^2+^ signaling and possibly gliotransmission as shown in the thalamus upon local *ex vivo* baclofen and GHB application (177).

As expected, the injection of the GAT inhibitor tiagabine in the thalamus enhances SWDs (178), while its injection in the somatosensory cortex suppresses SWDs, as does the injection of positive allosteric modulators of glutamate metabotropic receptors mGluR1 and mGluR5 in both somatosensory cortex and thalamus (178). A line of experimental evidence suggests that possibly all metabotropic glutamate receptors, including the mGluR2/3 and mGluR5 expressed on the astroglial membrane, are involved in SWDs through modulation of NMDA receptors and GABA uptake (178–182). Indeed, a subpopulation of astrocytes in the thalamus expresses mGluR5 and respond to cortico-thalamic glutamatergic afferents *via* intracellular Ca^2+^ oscillations (183). Therefore, it is very likely that astrocytes contribute to SWD phenotype by processing glutamate signaling. Astroglial glutamate transporters EAAT1 (GLutamate ASpartate Transporter, GLAST) and EAAT2 (GLutamate Transporter-1, GLT-1) (184, 185) as well as astroglial glutamine-glutamate-GABA cycle impairment (186, 187) have already been associated with the development of various forms of epileptic activities. GAERS rats display decreased protein expression of both astroglial GLT-1 and GLAST proteins before the development of absence seizures (188). Notably, GLAST is overexpressed at the mRNA level, possibly due to a compensatory mechanism of gene transcription (189). Moreover, excessive neuronal firing is known to induce astroglial swelling and subsequent glutamate release (190). This may add a further level of complexity in the already complex temporal firing dynamics of the thalamo-cortical neurons and NRT neurons both ictally and at interictal-to-ictal transitions (25). Although not yet proven in the context of SWDs, astrocytes possess the extraordinary capability of converting intensive glutamatergic neuronal activity into tonic inhibition, by coupling the glutamate/Na^+^ symport with the glutamine and GABA/Na^+^ symport (191). Notably, the only ATP expenditure associated with this process relies on the replenishment of the intracellular GABA storage since the driving force of the glutamine and GABA release is the re-establishment of the physiological Na^+^ homeostasis altered by the glutamate/Na^+^ symport. Finally, a comprehensive study of metabolic alterations in GAERS rats provides further insight into the cortical and thalamic astroglial contribution to the pathology of SWDs. Most strikingly, cortical astroglial metabolism and glutamine-glutamate-GABA cycle are enhanced in GAERS rats, leading to increased glutamate and glutamine levels and decreased GABA labeling (192). Interestingly, the expression of astroglial glutamate dehydrogenase is increased, in the cortex before the development of absence seizures and in the thalamus before and after the development of absence seizures, thus possibly leading to a decreased glutamate availability and a shift to the thalamic GABAergic inhibition fundamental for the generalization of SWDs (193). In line with this hypothesis, the intraperitoneal injection of branched-chain amino acids and α-ketoisocaproate pushing the chemical equilibrium toward the synthesis of glutamine led to decreased thalamic glutamate levels and the worsening of absence seizures (194). Moreover, a gain-of-function mutation of the glutamate dehydrogenase gene leading to aberrant glutamate availability and hyperammonemia has been associated with myoclonic absence epilepsy (195). Although further research in the field of absence epilepsy is still required, this evidence supports the role of astrocytic metabolism and glutamine-glutamate-GABA cycle in providing adequate energy supply and network homeostasis required for epileptic activity generation and propagation (94, 196).

*In situ* hybridization and Western blot analysis showed reduced levels of CB1 receptor mRNA and protein in the NRT and of the CB1 receptor in the thalamus of WAG/Rij rats at the protein level, thereby suggesting an impaired depolarization-induced CB1-mediated suppression of inhibition (197). Indeed, acute systemic injection of the synthetic CB1 receptor agonist WIN55,212-2 resulted in a transient reduction in SWDs frequency, however surprisingly followed by an increase in SWD duration in subchronic treatment (144, 197–199). Since the beneficial effects of the endocannabinoid ANA, previously described, last longer than the transient reduction in SWD frequency induced by the synthetic CB1 agonist and since ANA does indeed shorten SWDs, its mechanism of action is likely not only dependent on CB1 activation but a more complex molecular process (144).

The release of ATP through connexin and pannexin-1 hemichannels and the resulting spread of Ca^2+^ waves largely contribute to the astrocyte-mediated purinergic signaling in epilepsy (200). However, the net impact on the neural network is often context-dependent and may include the conversion of ATP into adenosine. Adenosine levels depend on extracellular ectonucleotidases as well as on the astroglial adenosine kinase (ADK) and its contribution encompasses antiepileptic A1 receptor-mediated as well as proepileptic A2 and A3 receptor-mediated effects (200–203). Once again, most research results have been derived from the analysis of convulsive seizures. Nevertheless, there is a number of evidence suggesting that purinergic signaling is altered in SWDs, too. To which extent this is related to astroglial contribution is still elusive. With respect to SWDs, GAERS rats show lower expression of A1 receptors in the NRT (204) and WAG/Rij rats are characterized by altered expression of A2A receptors in the somatosensory cortex, NRT and thalamus (205). Absence epileptic activity in WAG/Rij rats increases after activation of A2A receptors directly by the specific synthetic agonist 2-[4-(-2-carboxyethyl)-phenylamino]-5′-N-ethylcarboxamido-adenosine (CGS21680) (205) or indirectly after intraperitoneal injection of guanosine (206) as well as of adenosine (207). Conversely, acute caffeine administration, which is a mixed non-specific A1 and A2A receptor antagonist, reduced both amplitude and duration of SWDs in GAERS rats (208). However, the administration of the specific A1 antagonist 1,3.dipropyl-8-cyclopentylxanthine (DPCPX) in WAG/Rij rats had a proepileptic effect on SWDs (209). Notably, a duplication in the chromosomal region containing the gene coding for the extracellular catabolic enzyme adenosine deaminase was associated with a case of early-onset absence epilepsy, possibly leading to an impairment in adenosine homeostasis (210, 211).

The neuropeptide Y (NPY) released by thalamic neurons promotes phase-specific long-term depression of neuronal excitability in the NRT as well as in the thalamus itself and thus possibly contributing to thalamocortical synchronization and the altered dynamics of T-type Ca^2+^ channel-mediated bursting activity in the thalamic nuclei (212). Interestingly, valproic acid treatment increases thalamic levels of NPY mRNA in GAERS rats (213). Moreover, NPY intracerebroventricular injection as well as focal administration of NPY in the somatosensory cortex of GAERS rats had a strong antiepileptic effect mediated by the NPY receptor Y2 (214–216). This was confirmed by the analysis of specific NPY receptor knock-out mice (217, 218) and injection of the specific Y2 receptor agonist Ac[Leu (28, 31)] NPY24-36 and the specific Y2 receptor antagonist BIIE0246 in GAERS rats (215). Notably, viral overexpression of NPY as well as the mRNA of its receptor Y2, both in thalamus and somatosensory cortex of GAERS rats, reduced the number of seizures and the time spent in seizure activity (219). Since astrocytes produce (220) and release (221) NPY and express NPY receptors, including Y2 receptor (222, 223), one can imagine that astrocytes may play a role in NPY signaling in the pathophysiology of cortico-thalamo-cortical networks.

*Alterations of astroglial neurometabolic coupling and contribution to the glutamine-glutamate-GABA cycle may be at the basis of SWDs, possibly through enhanced metabolism and glutamate presentation to cortical neurons. Moreover, astroglial control of extracellular neurotransmitter level, based on the expression of glutamate and GABA transporters (EAATs and GATs, respectively) and receptors (both metabotropic and ionotropic) and direct and indirect release of glutamate and GABA, plays a fundamental role in maintaining the E/I balance in the cortex, thalamus and NRT. Astroglial ATP release and subsequent adenosine production seem to have context-dependent effects on neural excitability, but generally in line with observations derived from convulsive seizures pointing at an antiepileptic and proepileptic role of A1 and A2 receptors, respectively. Shifts in the E/I toward inhibition in the thalamus (possibly through altered endocannabinoid signaling) and toward excitation in the NRT and cortex have a pro-epileptic effect on SWDs. Unexpected net outcomes of pharmacological or genetic manipulation may be due to differential impact on different key nodes of the cortico-thalamo-cortical network and/or to astrocytic ability to both preserve and reverse the sign of the input signal*.

### The Classic Chicken and Egg Situation. Which Comes First: Astroglial Ca^2+^ Or Seizures?

Intracellular Ca^2+^ oscillations are one of the most studied indicators of astroglial activity and information coding mechanism at the core of the astroglial signaling cascade resulting, among others, in gliotransmission (86). In the context of convulsive epilepsy, excitotoxic spilling of glutamate, GABA and ATP resulting from excessive network activity as well as dying cells induce perturbation in astroglial Ca^2+^ signals (224, 225). Conversely, spontaneous as well as induced Ca^2+^ oscillations lead to gliotransmission thus influencing neuronal synchrony and E/I balance (226–234). Notably, astroglial Ca^2+^ elevations precede temporally neuronal engagement and their attenuation results in reduction of the epileptic activity in an *in vivo* model of temporal lobe epilepsy (TLE) (235). Moreover, astroglial Ca^2+^ activity is associated with spreading depolarization-mediated seizure termination (236). However, current research is far from understanding astroglial Ca^2+^ contribution to seizure generation, propagation, severity, and termination both in mechanistic and logical (sufficiency and/or necessity) terms. In particular, research on the contribution of astroglial Ca^2+^ signaling in seizure phenotype has not yet provided causative links to the SWD pathophysiology. Nevertheless, in the following paragraph we include some observations that encourage further research on the topic.

Thalamic astroglial networks display multi-cellular Ca^2+^ oscillations in absence of neuronal input and induce glutamate release and NMDA-receptor mediated long lasting inward currents in thalamocortical neurons as studied in acute brain slice preparations (226, 237). Thalamic astrocytes segregate into two groups: a first group with mGluR5-dependent and no voltage-dependent Ca^2+^ oscillations in response to cortico-thalamic activation, and a second group with no mGluR5- but voltage-dependent Ca^2+^ responses (183). Moreover, thalamic astroglial Ca^2+^ responses were recorded after acute *ex vivo* application of the weak GABA_B_ receptor agonist GHB (177), thus suggesting a putative role of astrocytes in the regulation of GABAergic signaling in the thalamus and possible in the phenomenology of SWDs. Notably, sustained GABA_B_ receptor activation led to a decrease in glutamate release from astrocytes (177). In addition, Ca^2+^ signaling and GABA seem to be connected since artificial inhibition of Ca^2+^ oscillation in striatal astrocytes leads to GAT3 functional upregulation and increased GABA uptake (238). Further evidence suggesting an integrative role of thalamic astrocytes in cortico-thalamic interactions comes from the observation that astroglial glutamate- and NMDA receptor- mediated slow inward currents (SICs) in the thalamo-cortical neurons are largely resistant to afferent cortico-thalamic inputs in their emergence but not in their frequency upon sustained input (239, 240). Moreover, cortico-thalamic glutamatergic input induced disinhibition of thalamo-cortical neurons through astroglial mGluR2 activation, Ca^2+^-dependent glutamate release and inhibition of presynaptic GABAergic projections from the NRT (241). In the NRT astrocytes also enhance GABA_A_ receptor signaling (242). Astrocyte-induced glutamate-mediated SICs of thalamo-cortical neurons seem to be dependent on extracellular glutamate levels, since exogenous exposure to the glutamate-mimetic D-aspartate increased the frequency of SICs (243). Although it is still unclear if abnormal or hypersynchronous astroglial Ca^2+^ signals could promote epileptiform network activity by itself, this evidence further supports an astroglial contribution to the propagation and self-sustain of seizure-like activity (244, 245).

The role of astrocytic Ca^2+^ signaling in epilepsy, and particularly in SWD-displaying epilepsies, is far from being understood. Yet, association studies on CAE and other idiopathic epileptic forms displaying SWDs as well as the evaluation of the genetic etiology of rodent absence epilepsy models point to a plethora of genes involved in voltage-gated Ca^2+^ channel signaling and G protein-coupled receptor signaling that is worth further assessment (7).

*Astroglia display spontaneous Ca*^*2+*^ *oscillations responsible for gliotransmission and homeostatic control of the E/I balance as well as network synchronicity. Moreover, astrocytes respond to physiological network activity and pathological neurotransmitter spilling and release from dying cells by Ca*^*2+*^ *elevations, typically further contributing to network priming, seizure initiation and progression. Conversely, Ca*^*2+*^ *signaling-induced gliotransmitter release and modulation of astroglial neurotransmitter receptors and transporters may underlie putative (or potential) anti-epileptic roles of Ca*^*2+*^ *signaling. Notably, astroglial Ca*^*2+*^ *signaling may also contribute to seizure suppression. To which extent this applies to SWDs is still unclear*.

### Reactive Astrogliosis and the Astrocyte-Derived Inflammatory Response May Contribute to the Pathology of SWDs

Astroglial proliferation and morphological, biochemical, and functional changes associated with epilepsy as well as with other neurodegenerative diseases are commonly referred to as reactive astrogliosis (246–248). The term is misleading since it implies that the pathological phenotype of astrocytes results from the epileptiform activity and oversees the possible causative role of astrocyte modifications in its genesis (249–251). In GAERS rats, cortical as well as thalamic astrocytes display enhanced expression of the glial fibrillary acidic protein (GFAP) even before the onset of absence seizures (193). Similarly, increased levels of GFAP expression can be found in adult WAG/Rij rats, though to a lesser extent than in GAERS rats (252). Astonishingly, the number of glial cells in the somatosensory cortex is significantly decreased (253). This suggests that biochemical and functional changes may contribute to a greater extent to the pathology of absence seizures than morphological alteration or that the latter involves qualitative astroglial reorganization, e.g., overlap of the astroglial processes, astroglial domain reorganization, structural and quantitative alteration of synaptic contacts or blood-brain barrier dysfunction. Notably, valproic acid diminishes the overlap of astroglial processes observed in correspondence of epileptic foci in several pathological models of convulsive seizures (254). Nevertheless, it is not clear if the same is happening in the pharmacodynamics of valproic acid in the context of SWDs. The same is true for the alterations of the blood-brain barrier (BBB) which have been associated with many pathological scenarios, including epilepsy (255), but whose role in SWDs has not been extensively addressed yet.

Pathological stimulation of astrocytes during convulsive epileptiform activity leads to astrocytic upregulation and release of proinflammatory cytokines, with IL-1β, Il-6, and TNFα as the most prominent ones. These factors, in turn, can induce astroglial dysfunction leading to, among others, increased glutamate release, decreased glutamate uptake, down-regulation of K_ir_4.1, AQP4, connexins, and glutamine synthetase as well as upregulation of adenosine kinase (256–258). IL-1β is induced in reactive astroglia in the somatosensory cortex (and not in other regions of the cortex) in adult GAERS rats with mature SWDs and interestingly also in some young GAERS in association with immature forms of SWDs (259). Furthermore, inhibition of IL-1β biosynthesis in adult GAERS reduced both the number as well as the duration of SWDs. Conversely, IL-1β intraperitoneal administration in WAG/Rij rats induced a significant increase in SWDs and worsened the proepileptic effects of the GABA reuptake inhibitor tiagabine (260). TNFα administration also aggravates SWDs but with kinetics incompatible with a direct effect and therefore possibly through *de novo* production of IL-1β itself. Moreover, before the onset of SWDs, young WAG/Rij rats showed increased TNFα blood levels, which gradually decreased with age and returned to physiological levels in adult rats displaying mature SWDs, thus possibly suggesting a neuroprotective role of TNFα (260). The precise mechanism of TNFα action in this scenario is not clear, although it is known that TNFα reduces astroglial glutamate uptake and decreases neuronal GABA_A_ receptor expression (261, 262). Notably, IL-1β-, TNFα-, and IL-6-inducing lipopolysaccharide (LPS) injection in WAG/Rij also promoted SWDs and the increase in the latter was prevented by blocking the inflammatory response with indomethacin (263, 264) as well as blockers of the mTOR pathway (265, 266). Similarly, LPS effects on SWDs were later confirmed in GAERS rats (267). Although IL-1β is believed to increase the levels of glutamate, co-administration of LPS and the NMDA receptor antagonist D-(-)-2-Amino-5-phosphonopentanoic acid (AP-5) did not counteract LPS effects as expected, but conversely prolonged them (264). Recently, it has been reported that IL-6 receptor (IL-6R) blockage *via* tocilizumab (a humanized monoclonal antibody against IL-6R) reduces SWDs in WAG/Rij rats and inhibits their LPS-induced worsening (268). In line with that, human CAE is known to be associated with detectable levels of IL-6 and IL-8 in the cerebrospinal fluid (269) and treatment with valproic acid reduces IL-6 serum levels in children with tonic-clonic generalized seizures (270).

*Several lines of evidence support a role for a direct contribution of pro-inflammatory cytokines in the genesis and worsening of SWDs. Notably, astroglial alterations and cytokine release precede SWD onset, although it cannot be excluded that these cell responses may be due to subclinical epileptiform activities or genetic predispositions. IL-1*β*, IL-6 and TNF*α *may contribute to the pathology of SWDs, possibly through impaired K*^+^ *clearance, glutamine-glutamate-GABA cycle, adenosine metabolism, gliotransmission, and neurotransmitter reuptake. Other morphological alterations, such as astroglial overlap, connectivity, and synaptic coverage, may play a role as well*.

## Absence Seizures and Nrem Sleep: Two Sides of the Same Coin?

**Figure 4:**
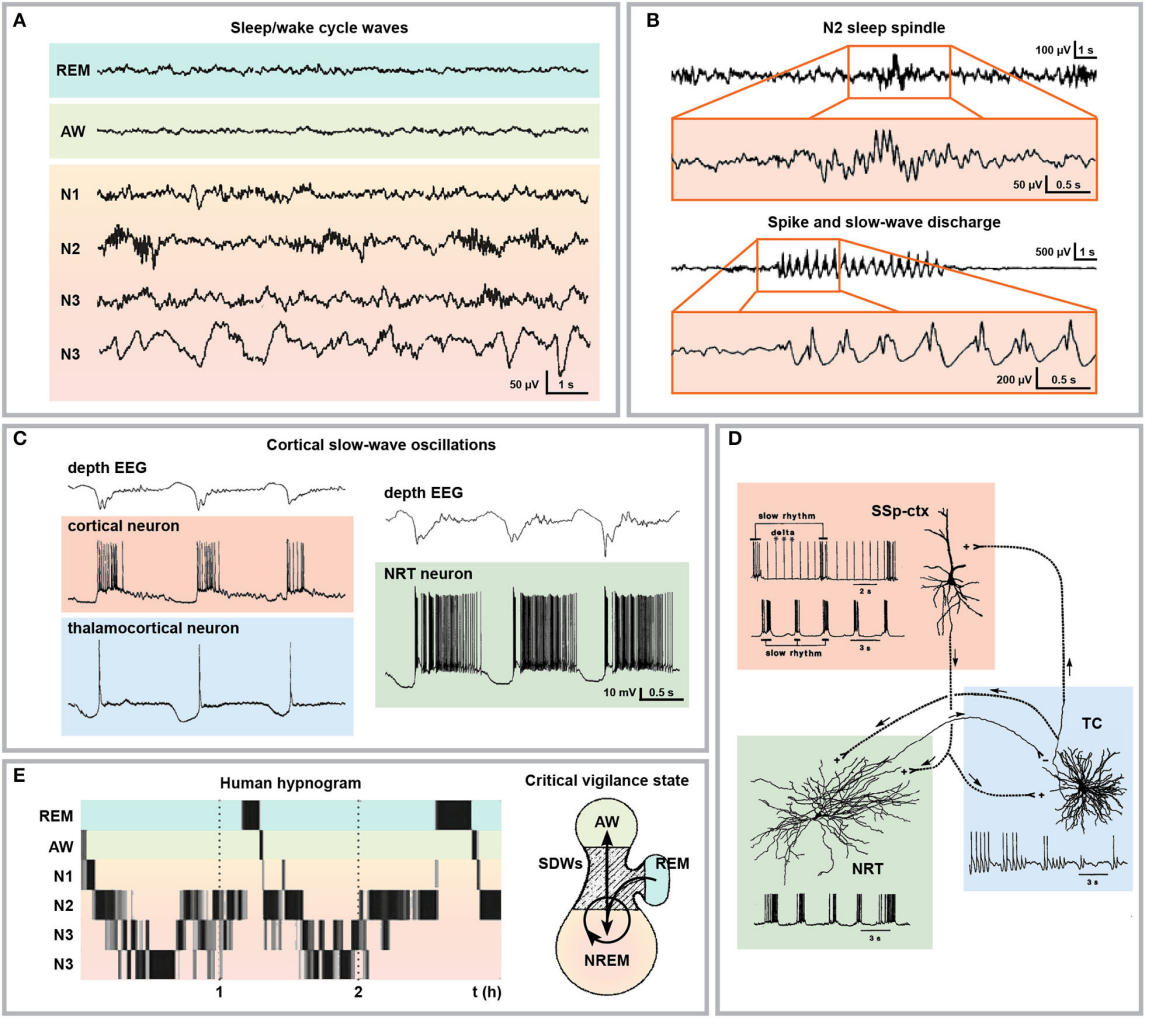
Electrophysiological and cellular bases of sleep and SWD relationship. **(A)** Representative human electroencephalographical wave recordings during wakefulness (AW), REM sleep and different NREM sleep stages (N1, passive wakefulness or light sleep; N2, light slow-wave sleep; N3, deep slow-wave sleep). **(B)** Sleep spindle typically occurring during stage N2 of NREM sleep with respective magnification and comparison with SWDs. **(C)** Depth cortical EEG recording displaying cortical slow wave oscillations (upper traces) and time-matched intracellular recordings from cortical, thalamocortical, and NRT neurons with typical burst firing activity. **(D)** Schematic representation of the cellular and electrical components of cortico-thalamo-cortical oscillations. **(E)** Human hypnogram displaying two typical sleep cycles characterized by the succession of the NREM sleep stages followed by one episode of REM sleep (left) and schematic representation of the critical vigilance level (hatched area) promoting SWD occurrence during transitions between NREM and wakefulness, between NREM stages and from (but not to) REM sleep (right). Modified from **(A)**, (271); **(B)**, (272), **(C)**, (273); **(D)**, (274); **(E)**, (271, 275).

The cortico-thalamo-cortical network processes behaviourally relevant internal and external information and determines vigilance states as well as neuronal network oscillation during sleep (Figure 4A), thus playing a fundamental role in both physiology and pathology (25, 276–281). Several lines of evidence suggest that epilepsy and sleep are strongly related (282). Notably, various forms of epilepsy display different incidences across the 24 h sleep/wake cycle and among different sleep stages, possibly due to specific seizure susceptibility dependent on brain excitability and network engagement (283–285).

Till recently, SWDs were considered the pathological transformation of sleep spindles (also known also thalamocortical spindles) occurring during stage II NREM sleep (Figure 4B) (272, 286, 287). This concept was mainly supported by studies on the temporal coincidence of sleep spindles and SWDs (288, 289) and on the progressive transformation of sleep spindles into SWDs observed after intramuscular injection of penicillin in cats (24, 290). Indeed, to some extent both sleep spindles and SWDs share some anatomical, cellular and molecular mechanisms (291) and they are functionally correlated (292, 293). However, the identification of SWDs as pathological transitions from sleep spindles has been recently challenged (294–296), in favor of a predominant role of cortical slow (<1 Hz) oscillations alternating active (Up) and silent (Down) cortical activity and concomitantly occurring during NREM sleep (Figures 4C,D) (273, 274, 297–301). SWDs largely arise in a specific critical vigilance window in correspondence with passive wakefulness, transitions to NREM slow-wave sleep as well as during transitions between internal substages of NREM sleep (stage I to III; N1: light sleep or passive wakefulness, N2: light slow-wave sleep and N3: deep slow-wave sleep, respectively) (Figure 4E). Moreover, SWDs are disrupted by arousing stimuli and do not transition to REM sleep directly (38, 275, 302–308), thus suggesting that absence seizures prefer low and shifting-vigilance periods during superficial slow-wave NREM sleep (282). With respect to the incidence of seizures across the 24 h cycle, the distribution of generalized SWDs is still under debate. Seizures originating in the frontal lobe (as absence seizures are currently believed to be) are more frequent at night and in sleep (309, 310). Conversely though, dialeptic and atonic seizures occur more often during daytime (310). Generalized pediatric seizures, including absence seizures, were reported to occur predominantly during wakefulness (311, 312) but were restricted to NREM sleep stages I and II when occurring during the night and were almost absent during REM sleep (313, 314). Moreover, a study on idiopathic generalized epilepsies including CAE and other SWD-displaying epilepsies showed that interictal epileptic discharges are more frequent during NREM sleep and occur mainly at sleep onset (315). In WAG/Rij rats, SWDs are most frequent in the beginning of the dark phase and are at their minimum frequency at the onset of the light phase (316, 317). If rats are artificially kept in dim light (thus disrupting the 12:12 light-dark cycle), SWDs still display 24 h cyclicity, proving its endogenous rhythmicity, but the cycle is desynchronized with respect to the rhythm of the general motor activity, thus suggesting that the mechanism governing SWDs and sleep/wake cycles are different (317, 318). Interestingly, after an artificial shifting in the light-dark cycle, SWDs resynchronized at the same speed of light slow-wave speed in comparison with both REM and deep slow-wave sleep (319), pointing at the existence of a common circadian mechanism governing SWDs and light slow-wave sleep. Taken together, it seems that conditions associated with highly desynchronized (active wakefulness and REM sleep) and highly synchronized (deep slow-wave sleep) cortical activity tend to inhibit SWDs. In line with this hypothesis, the anti-absence molecule uridine (320) impacts sleep architecture by fragmenting sleep, thus increasing the frequency of NREM-REM transitions and by inducing preferentially REM sleep (321).

With respect to the putative interdependency of SWDs and NREM sleep waves, it was reported that sleep deprivation has a proepileptic effect on both humans (322–325) and rodents (304, 305, 326, 327). On the other side, epilepsy is associated with sleep alterations, including sleep fragmentation, day-time drowsiness and difficulties in sleep initiation (328). To date, the field still lacks a systematic clinical study on the effect of absence epilepsy on sleep. Nevertheless, it was shown in WAG/Rij rats that SWDs disrupt NREM sleep and sleep architecture (329). Moreover, epilepsy-induced sleep alterations depend on the timing of the epileptiform activity. In a time-controlled kindling epileptic model in rats, seizure induction at the transition from light to dark (*zeitgeber* time (ZT) 0) and from dark to light (ZT13) altered both NREM and REM duration without affecting sleep/wake cycles and the sole seizure induction at ZT13 induced increased levels of IL-1 and increased NREM sleep specifically (330). Interestingly, both IL-1β and TNFα increase the amount of NREM sleep (331), which could contribute to the increase of SWD number after LPS injection (263, 264) by an increased state of passive awareness and slow-wave sleep. Indeed, *in silico* meta-analysis of differentially expressed proteins from the fronto-parietal cortex and thalamus of LPS-treated WAG/Rij rats supports this scenario, given the overrepresentation of proteins associated with sleep regulation (332). Moreover, the pathological activation of the mTOR pathway involved in LPS-induced increase in SWDs (265, 266) is responsible for the upregulation of the core clock gene product aryl hydrocarbon receptor nuclear translocator-like protein 1 (ARNTL), also known as brain and muscle ARNT-Like 1 (BMAL1), as observed in a model of tuberous sclerosis complex, a neurological disorder displaying epileptic activity (333). BMAL1 not only is a key component of both circadian and sleep/wake cycles (334) as well as susceptibility to seizures and epilepsy (335), but it is also at the basis of cell-autonomous circadian clock of astrocytes (336, 337).

*SWD occurrence varies during the sleep/wake cycle with respect to both sleep and vigilance states. Notably, SWDs peak in correspondence of low and shifting-vigilance periods during superficial slow-wave NREM sleep and are underrepresented during active wakefulness and REM sleep. Resynchronization studies after shifting in the light-dark cycle suggest a common circadian mechanism governing SWDs and NREM sleep. Moreover, even though the nature and the causative link between the two are far from being clearly understood, SWDs and NREM sleep are similarly and consistently altered by a number of pathological and pharmacological alterations*.

### Both Sleep Architecture and Sleep/Wake Cycle Are Shaped by Astroglial Activity

**Figure 5:**
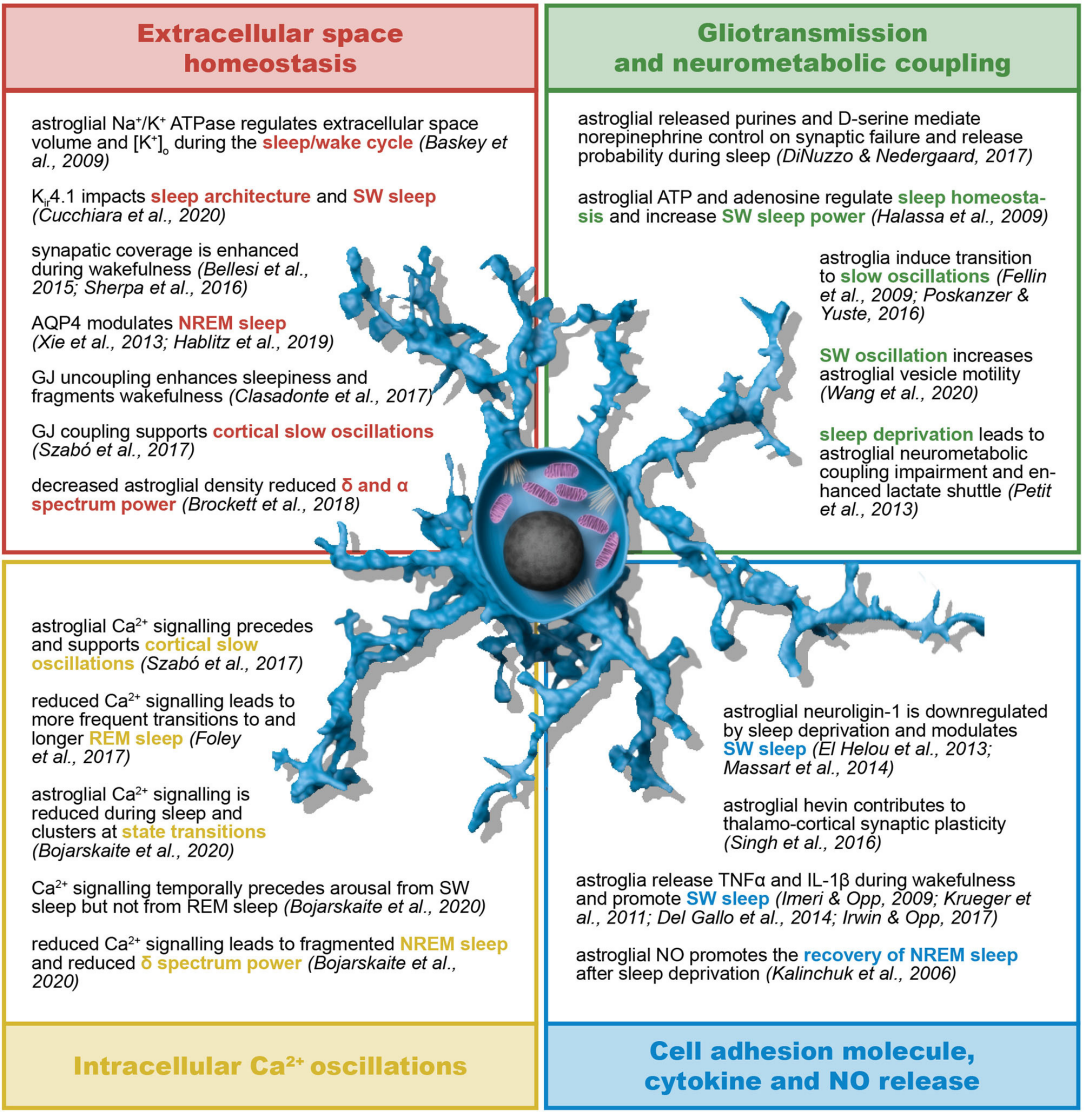
Astroglial role in sleep/wake cycle and sleep architecture. Astrocytes contribute to sleep homeostasis in terms of both sleep/wake cycle regulation and sleep architecture and dynamics. The astroglial regulation of sleep relies on their control of extracellular K^+^ concentration, interstitial fluid exchanges and spatial buffering through gap junctions. Moreover, astroglia support and influence neural activity by means of their neurometabolic coupling to neurons, intracellular Ca^2+^ oscillations, gliotransmission, and release of, among other, cell adhesion molecules, cytokines and nitric oxide. AQP4, aquaporin-4; ATP, adenosine triphosphate; GJ, gap junction; IL-1β, interleukin-1β; K_ir_, inward rectifying K^+^ channels; NO, nitric oxide; (N)REM, (non-) rapid eye movement; SW, slow wave; TNFα, tumor necrosis factor α.

Given the fact that both SWDs and NREM recruit the cortico-thalamo-cortical network, further insights into the role of astrocytes in the pathophysiology of SWDs may derive from evidence in their contribution to sleep (particularly sleep architecture and sleep/wake cycle) (Figure 5) as well as the circadian cycle. Astroglial impact on circadian clock mechanisms generated in deep structures may contribute to epilepsy in non-intuitive ways (335). In the hypothalamic primary timekeeping center, the suprachiasmatic nucleus (SCN), astrocyte-derived glutamate inhibits neuronal firing through presynaptic NMDA receptors specifically during the night (338). Moreover, astrocytes release adenosine in a CB1 receptor- and intracellular Ca^2+^ signaling-dependent manner and induce the disinhibition of SCN neurons (339). WAG/Rij rats are also characterized by astrogliosis and impaired GABAergic transmission in the thalamic intergeniculate leaflet, which coordinates inputs from the retina and outputs to the SCN (340). The research on the role of astrocytes on timekeeping is still at its early days, but several lines of evidence support astroglial contribution to sleep, namely the modulation of sleep homeostasis, sleep pressure, vigilance states and sleep-dependent cognitive function, brain energetics and network metabolic supply, network excitability and sleep-associated waste clearance (341, 342).

The sleep/wake cycle is associated with changes in interstitial fluid and of the ionic composition with increased extracellular space and decreased interstitial K^+^ concentration during sleep (343–345), a process that involves norepinephrine-mediated inhibition of the astroglial Na^+^/K^+^ pump during wakefulness (346). This process is responsible for widespread neuronal hyperpolarization and decreased firing rate particularly during NREM sleep (347, 348). In parallel, it was recently reported that children with an autism-associated epilepsy phenotype carrying a gain-of-function mutation in the K_ir_4.1 coding KCHJ10 gene display abnormal slow-wave sleep with a significantly longer slow-wave period (349). Norepinephrine induces astroglial process elongation and astroglial synaptic coverage during wakefulness (345, 350). Conversely, decreased levels of norepinephrine may be responsible for reduction of direct and indirect astroglial release of ATP/adenosine and D-serine, thus contributing to the overall decreased synaptic failure and increased release probability during sleep. Interestingly, norepinephrine level is particularly low during NREM sleep, due to suppression of noradrenergic neuronal firing (351).

Astroglial-dependent cerebrospinal fluid (CSF) flow is responsible for waste and interstitial fluid clearance during sleep (352) and inward flow of CSF through astroglial AQP4 occurs mainly during NREM sleep (343, 353). The CSF flow is under circadian control mediated by changes of AQP4 polarization (354). Recently, a haplotype of AQP4 carrying several single nucleotide polymorphisms (SNPs), among which some associated with reduced AQP4 expression, has been linked to altered slow-wave NREM sleep modulation (355). Moreover, astroglial gap junction coupling is likely to contribute to the regulation of the sleep/wake by means of modulation of both CSF flow and waste clearance. To date, most studies addressing GJ coupling in astrocytes are focused on the altered metabolite trafficking (namely glucose and lactate) resulting from GJ manipulation that impairs the fundamental role of astrocytes in synaptic energy support and brain energy metabolism (356). Astrocyte-specific conditional knock-out of Cx43 in mice resulted in enhanced sleepiness, fragmented wakefulness, and impaired neuromodulation of the sleep/wake cycle (357). Conversely, astroglial neurometabolic coupling impairment results from sleep deprivation that leads to astroglial upregulation of the transporters GLUT1, GLT1, the Na^+^/K^+^ pump as well as other components of the astrocyte-neuron lactate shuttle (358). Sleep deprivation could therefore possibly contribute to increased network activity also through enhanced lactate delivery to neurons as suggested by the fact that the anticonvulsant stiripentol is a lactate dehydrogenase inhibitor (359) in addition to being a positive allosteric modulator of GABA_A_ receptors (360).

Impairment of astroglial exocytosis and gliotransmission using cell-specific expression of dnSNARE results in reduced tonic A1R adenosinergic signaling, altered sleep homeostasis and reduced slow-wave power, and reduced sleep pressure in mice (361), as confirmed by previous evidence suggesting the role of A1 receptors in augmented sleep pressure (362–364). The same genetic manipulation suppressed the LPS-induced increase in slow-wave power during NREM sleep, proving the astroglial contribution to inflammatory-derived increased sleep pressure (365). Although the role of A2 receptor (A2R) activation by astroglial adenosine is still controversial, A2Rs may play a role in sleep homeostasis through activation of A2AR-expressing neurons in the nucleus accumbens core involved in the induction of slow-wave sleep (366). In the cortex, altered gliotransmission resulted in reduced neuronal NMDA receptor activity and reduced slow oscillations (367), whereas astroglial specific activation induced neuronal transition to slow oscillations (368). *In vivo* characterization of Ca^2+^ signaling in both rat cortical astrocytes and neurons revealed that astroglial synchronized activity reliably precedes neuronal oscillations and that both astrocyte uncoupling and intracellular Ca^2+^ chelation reduced the fraction of astrocytes and neurons involved in the cortical slow waves. Remarkably, neurons closer to active astrocytes were more likely involved in the oscillations (369). Recently, simultaneously recording of BOLD fMRI and astroglial Ca^2+^ signaling in anesthetized rats revealed that a fraction of intrinsic cortical Ca^2+^ signals were associated with reduced EEG power and negative fMRI signal throughout the cortex (correlated with decreased neuronal activity) and that increased activity in the thalamus specifically preceded these signals (370). Conversely, reduction in the density of cortical astrocytes in the medial prefrontal cortex (and therefore putatively in their connectivity) has been linked to a decrease in δ (0.5–4 Hz) and α (8–12 Hz) spectrum power (371). Moreover, mice overexpressing an astrocyte-specific inositol triphosphate (IP_3_) phosphatase, and therefore displaying reduced IP_3_-dependent Ca^2+^ activity, spent more time in REM sleep and revealed more transitions to REM sleep from passive wakefulness (372). In line with that, mice lacking the inositol 1,4,5-triphosphate receptor type 2 exhibit reduced Ca^2+^ signaling, a more fragmented and shorter NREM sleep associated with decreased δ spectrum power and more frequent microarousals (373). Remarkably, astroglial intracellular Ca^2+^ increases precede the transition from NREM sleep to wakefulness but follow arousal from REM sleep (373), thus suggesting that astroglia could mediate norepinephrine-induced arousal from NREM sleep (374). Furthermore, it has been recently shown that *in vitro* application of an oscillatory electric field specifically in the slow-wave range (and not at higher frequencies) increased astroglial synaptic vesicle mobility (375), thus suggesting a positive feedback mechanism for slow-wave state perpetuation. Taken together, these data suggest that astroglial connectivity, astroglial Ca^2+^ waves and gliotransmitter release may initiate and/or support the initiation of cortical slow wave oscillations or favor this transition over others (e.g., passive wakefulness-REM).

Another piece of evidence supporting the contribution of astroglial synaptic plasticity in the modulation of sleep derives from studies focused on the extracellular matrix components in the synaptic cleft. In the mouse forebrain, sleep pressure after sleep deprivation decreased the expression of astroglial neuroligin-1 (376), a cell adhesion molecule binding to presynaptic neurexins (377, 378). Conversely, neuroligin-1 knock-out mice have increased slow-wave sleep and enhanced synchrony during sleep (379). Astrocytes contribute to glutamatergic synaptic plasticity of thalamo-cortical synapses through secretion of hevin, a synaptogenic protein inducing the interaction between non-canonical synaptic partners including neuroligin-1 (380). Knock-out mice lacking another member of the neuroligin family, neuroligin-2, develop SWDs and behavioral arrests, a phenotype blocked by ethosuximide and attenuated by expression of neuroligin-2 selectively in the thalamic neurons or optogenetic activation of GABAergic projections from the NRT (381). This could be due to an interaction with GABA_A_ receptors as suggested by previous studies on sleep-deprived mice (382). Mice with a missense mutation in neuroligin-3 exhibit an altered EEG power spectrum (383). Moreover, variations in the copy number of the gene encoding the postsynaptic scaffolding protein Shank3, which interacts with both neuroligin and glutamate receptors thus regulating synaptic plasticity (384, 385), have been linked to epileptiform activity specifically arising during slow-wave sleep (386). Shank3 loss-of-function mutations have been associated with several different epileptic forms but most commonly with atypical absence seizures (387).

Finally, as mentioned already above, inflammation and sleep are tightly intertwined. TNFα and IL-1β increase during wakefulness and decrease during sleep both at mRNA and protein level (388–390) and their systemic injection selectively increase slow-wave sleep (389, 391). Mice lacking IL-1β and TNFα receptors are characterized by less slow-wave and REM sleep (392, 393). Notably, agonists of both P2X- and P2Y-type purinergic receptors are known to activate the astroglial release of TNFα and IL-1β (394) and pannexin-1 knock-out mice with impaired ATP release display altered slow-wave sleep (395). Interestingly, unilateral cortical TNFα injection induces state-specific EEG asymmetries during NREM sleep (396) and ipsilateral increase in the number of IL-1β positive cells (mainly astrocytes) in the cortex, the NRT and in the thalamic nuclei (397). Along with TNFα and IL-1β, reactive astroglia also produce nitric oxide (NO) *via* inducible NO synthetase (iNOS) (398, 399) and NO has been linked to the pathology of epilepsy (400, 401). NO affects both NREM and REM sleep (402, 403) but inhibiting iNOS in sleep-deprived mice specifically impairs NREM recovery, whereas inhibiting neuronal NOS (nNOS) impairs the recovery of REM sleep (404), thus suggesting a specific role of astrocyte in NREM physiology.

*Astroglial homeostatic control of the extracellular space and synaptic transmission through K*^+^ *clearance mediates widespread neuronal hyperpolarization and decreased firing activity during sleep and impacts NREM sleep architecture. Astroglial regulation of the extracellular volume controls both waste and cerebrospinal fluid clearance essential for correct functioning of the brain and relies on AQP4-mediated water influx and GJ coupling. Adenosine released by astrocytes governs sleep homeostasis, sleep pressure and slow-wave power and astroglial gliotransmission contributes to neuronal slow oscillations in the cortex. Moreover, artificial lowering of astroglial Ca*^*2+*^ *oscillations and connectivity leads to reduced slow-wave power and relative shortening and fragmentation of NREM sleep. Finally, astroglial TNF*α*, IL-1*β*, and NO are likely to impact sleep architecture by selective increase of slow-wave sleep. These findings support the fact that astroglia play a fundamental role in the physiology of NREM sleep and therefore represent a promising target to study pathophysiological alterations inducing or sustaining the abnormal recruitment of the cortico-thalamo-cortical network during SWDs*.

## Conclusion

From a clinical, social and human point of view, epilepsy is probably one of the most heterogeneous neurological diseases. This variability partially relies on the fact that epilepsy may originate from a plethora of different conditions, among others traumatic injury, stroke, CNS infections or inflammation, brain tumor, genetic predisposition, and drug or alcohol abuse. In addition, in six out of 10 epilepsy cases the pathological origin is unknown. Yet, from a scientific point of view, epilepsy can ultimately be reduced to a local imbalance of excitation and inhibition and altered synchrony and functioning of neural networks in the brain. The heterogeneity of pathological outcomes associated with epilepsy arises from the variety and complexity of functions carried out by the human brain and the multiple layers of fine-tuning that each of them requires for reliable physiological functioning of the electrical activity in neural circuits. Given the existence of common molecular and cellular mechanisms at the basis of epilepsy and their nature as pathological transitions of altered physiological processes, both epilepsy research and clinical treatment benefit from the understanding of the inner functioning of neural networks.

Since spike and slow-wave discharges (SWDs) share some key anatomical and functional physiological brain oscillations naturally occurring during slow-wave sleep, absence seizure research could advance our understanding of both epilepsy and healthy brain mechanisms. In this review, we collected evidence supporting the functional and mechanistic relationship between slow-wave sleep and SWDs, thus providing insights into network alterations that contribute to the pathology of SWDs. Moreover, proving and characterizing the interdependency between epilepsy, sleep architecture and sleep/wake cycles possess an undeniable therapeutic value, since sleep is a pre-existing condition affecting any treatment outcome and efficacy.

We focused our attention on the role of astrocytes in the physiology of sleep and in their putative pathophysiological contribution to SWDs. Astroglial control on extracellular homeostasis in terms of ionic composition, volume regulation and transmitter clearance, astroglial connectivity, Ca^2+^ signaling and gliotransmission as well as cytokine release are hallmarks of astroglial function for physiological brain performance and were addressed in the context of SWDs and sleep research. Please note that it is insufficient and underestimating of the system complexity to label the astrocytic contribution to neural homeostasis as exclusively anti- or pro-epileptic. Many astroglial mechanisms may be beneficial or detrimental with respect to different forms of epilepsy, not to mention different network connectivities and states. Current research on astroglial contribution to epileptic brain functioning mostly relies on studies focused on convulsive seizures, possibly due to their lower remission rate and their clinical symptoms which appear more obvious and life threatening. Nevertheless, some clues suggest the mechanisms governing network excitability and synchrony may have a role in SWDs, too. This work was not intended to be and is far from being comprehensive neither of the role of astroglia in epilepsy nor of their contribution to sleep homeostasis and architecture but provides with significant associations in the tripartite synapse engaging astroglia, epilepsy and sleep in the context of the pathophysiology of cortico-thalamo-cortical oscillations. Understanding how astroglia contribute to the mechanisms underlying slow-wave sleep and how these are altered in pathology could possibly shine light on new therapeutical targets for a plethora of epileptic forms displaying SWDs, among which absence epilepsy, a condition that still affects 50 million people worldwide and is pharmacoresistant in almost one third of those.

## Author Contributions

DG screened the literature, conceptualized the review focus, wrote the first draft, designed and realized the figures, and finalized the manuscript. AS and FK contributed to figure conceptualization, reviewed and finalized the manuscript. All authors approved on the final version of the manuscript.

## Conflict of Interest

The authors declare that the research was conducted in the absence of any commercial or financial relationships that could be construed as a potential conflict of interest.

## References

[1] Huff J Murr N. Seizure. In: StatPearls. Treasure Island, FL: StatPearls Publishing (2021). Available online at: https://www.ncbi.nlm.nih.gov/books/NBK430765/

[2] Fisher RS Acevedo C Arzimanoglou A Bogacz A Cross JH Elger CE. ILAE official report: a practical clinical definition of epilepsy. Epilepsia. (2014) 55:475–82. 10.1111/epi.12550 24730690

[3] Scheffer IE Berkovic S Capovilla G Connolly MB French J Guilhoto L. ILAE classification of the epilepsies: position paper of the ILAE Commission for Classification and Terminology. Epilepsia. (2017) 58:512–21. 10.1111/epi.13709 28276062 PMC5386840

[4] Panayiotopoulos CP. Typical absence seizures and related epileptic syndromes: assessment of current state and directions for future research. Epilepsia. (2008) 49:2131–9. 10.1111/j.1528-1167.2008.01777.x 19049569

[5] Guo JN Kim R Chen Y Negishi M Jhun S Weiss S. Impaired consciousness in patients with absence seizures investigated by functional MRI, EEG, and behavioural measures: a cross-sectional study. Lancet Neurol. (2016) 15:1336–45. 10.1016/S1474-4422(16)30295-2 27839650 PMC5504428

[6] Panayiotopoulos CP Koutroumanidis M Giannakodimos S Agathonikou A. Idiopathic generalised epilepsy in adults manifested by phantom absences, generalised tonic-clonic seizures, and frequent absence status. J Neurol Neurosurg Psychiatry. (1997) 63:622–7. 10.1136/jnnp.63.5.622 9408104 PMC2169820

[7] Crunelli V Leresche N. Childhood absence epilepsy: genes, channels, neurons and networks. Nat Rev Neurosci. (2002) 3:371–82. 10.1038/nrn811 11988776

[8] Blumenfeld H. Consciousness and epilepsy: why are patients with absence seizures absent? Prog Brain Res. (2005) 150:271–86. 10.1016/S0079-6123(05)50020-7 16186030 PMC3153469

[9] Camfield C Camfield P. Management guidelines for children with idiopathic generalized epilepsy. Epilepsia. (2005) 46(Suppl.9):112–6. 10.1111/j.1528-1167.2005.00322.x 16302884

[10] Gardiner M. Genetics of idiopathic generalized epilepsies. Epilepsia. (2005) 46(Suppl.9):15–20. 10.1111/j.1528-1167.2005.00310.x 16302872

[11] Matricardi S Verrotti A Chiarelli F Cerminara C Curatolo P. Current advances in childhood absence epilepsy. Pediatr Neurol. (2014) 50:205–12. 10.1016/j.pediatrneurol.2013.10.009 24530152

[12] Berg AT Levy SR Testa FM Blumenfeld H. Long-term seizure remission in childhood absence epilepsy: might initial treatment matter? Epilepsia. (2014) 55:551–7. 10.1111/epi.12551 24512528 PMC3999182

[13] Glauser TA Cnaan A Shinnar S Hirtz DG Dlugos D Masur D. Ethosuximide, valproic acid, and lamotrigine in childhood absence epilepsy: initial monotherapy outcomes at 12 months. Epilepsia. (2013) 54:141–55. 10.1111/epi.12028 23167925 PMC3538883

[14] Masur D Shinnar S Cnaan A Shinnar RC Clark P Wang J. Pretreatment cognitive deficits and treatment effects on attention in childhood absence epilepsy. Neurology. (2013) 81:1572–80. 10.1212/WNL.0b013e3182a9f3ca 24089388 PMC3806916

[15] Holmes GL Noebels JL. The epilepsy spectrum: targeting future research challenges. Cold Spring Harb Perspect Med. (2016) 6:a028043. 10.1101/cshperspect.a028043 27371672 PMC4930917

[16] Cnaan A Shinnar S Arya R Adamson PC Clark PO Dlugos D. Second monotherapy in childhood absence epilepsy. Neurology. (2017) 88:182–90. 10.1212/WNL.0000000000003480 27986874 PMC5224720

[17] Crunelli V Lorincz ML McCafferty C Lambert RC Leresche N Di Giovanni G. Clinical and experimental insight into pathophysiology, comorbidity and therapy of absence seizures. Brain. (2020) 143:2341–68. 10.1093/brain/awaa072 32437558 PMC7447525

[18] Shi Q Zhang T Miao A Sun J Sun Y Chen Q. Differences between interictal and ictal generalized spike-wave discharges in childhood absence epilepsy: a MEG study. Front Neurol. (2019) 10:1359. 10.3389/fneur.2019.01359 32038453 PMC6992575

[19] Cerminara C Coniglio A El-Malhany N Casarelli L Curatolo P. Two epileptic syndromes, one brain: childhood absence epilepsy and benign childhood epilepsy with centrotemporal spikes. Seizure. (2012) 21:70–4. 10.1016/j.seizure.2011.09.005 22000044

[20] Coenen AM Van Luijtelaar EL. Genetic animal models for absence epilepsy: a review of the WAG/Rij strain of rats. Behav Genet. (2003) 33:635–55. 10.1023/A:1026179013847 14574120

[21] Panayiotopoulos CP. Treatment of typical absence seizures and related epileptic syndromes. Paediatr Drugs. (2001) 3:379–403. 10.2165/00128072-200103050-00006 11393330

[22] Bai X Vestal M Berman R Negishi M Spann M Vega C. Dynamic time course of typical childhood absence seizures: EEG, behavior, and functional magnetic resonance imaging. J Neurosci. (2010) 30:5884–93. 10.1523/JNEUROSCI.5101-09.2010 20427649 PMC2946206

[23] Tangwiriyasakul C Perani S Centeno M Yaakub SN Abela E Carmichael DW. Dynamic brain network states in human generalized spike-wave discharges. Brain. (2018) 141:2981–94. 10.1093/brain/awy223 30169608 PMC6158757

[24] von Krosigk M Bal T McCormick DA. Cellular mechanisms of a synchronized oscillation in the thalamus. Science. (1993) 261:361–4. 10.1126/science.8392750 8392750

[25] McCafferty C David F Venzi M Lorincz ML Delicata F Atherton Z. Cortical drive and thalamic feed-forward inhibition control thalamic output synchrony during absence seizures. Nat Neurosci. (2018) 21:744–56. 10.1038/s41593-018-0130-4 29662216 PMC6278913

[26] Meyer J Maheshwari A Noebels J Smirnakis S. Asynchronous suppression of visual cortex during absence seizures in stargazer mice. Nat Commun. (2018) 9:1938. 10.1038/s41467-018-04349-8 29769525 PMC5955878

[27] Panayiotopoulos CP. Typical absence seizures and their treatment. Arch Dis Child. (1999) 81:351–5. 10.1136/adc.81.4.351 10490445 PMC1718096

[28] Blumenfeld H. Cellular and network mechanisms of spike-wave seizures. Epilepsia. (2005) 46(Suppl.9):21–33. 10.1111/j.1528-1167.2005.00311.x 16302873

[29] Sitnikova E van Luijtelaar G. Electroencephalographic characterization of spike-wave discharges in cortex and thalamus in WAG/Rij rats. Epilepsia. (2007) 48:2296–311. 10.1111/j.1528-1167.2007.01250.x 18196621

[30] Terlau J Yang JW Khastkhodaei Z Seidenbecher T Luhmann HJ Pape HC. Spike-wave discharges in absence epilepsy: segregation of electrographic components reveals distinct pathways of seizure activity. J Physiol. (2020) 598:2397–414. 10.1113/JP279483 32144956

[31] Aghakhani Y Bagshaw AP Bénar CG Hawco C Andermann F Dubeau F. fMRI activation during spike and wave discharges in idiopathic generalized epilepsy. Brain. (2004) 127:1127–44. 10.1093/brain/awh136 15033899

[32] Gotman J Grova C Bagshaw A Kobayashi E Aghakhani Y Dubeau F. Generalized epileptic discharges show thalamocortical activation and suspension of the default state of the brain. Proc Natl Acad Sci USA. (2005) 102:15236–40. 10.1073/pnas.0504935102 16217042 PMC1257704

[33] Hamandi K Salek-Haddadi A Laufs H Liston A Friston K Fish DR. EEG-fMRI of idiopathic and secondarily generalized epilepsies. Neuroimage. (2006) 31:1700–10. 10.1016/j.neuroimage.2006.02.016 16624589

[34] Moeller F Siebner HR Wolff S Muhle H Granert O Jansen O. Simultaneous EEG-fMRI in drug-naive children with newly diagnosed absence epilepsy. Epilepsia. (2008) 49:1510–9. 10.1111/j.1528-1167.2008.01626.x 18435752

[35] Moeller F LeVan P Muhle H Stephani U Dubeau F Siniatchkin M. Absence seizures: individual patterns revealed by EEG-fMRI. Epilepsia. (2010) 51:2000–10. 10.1111/j.1528-1167.2010.02698.x 20726875 PMC3769289

[36] Bai X Guo J Killory B Vestal M Berman R Negishi M. Resting functional connectivity between the hemispheres in childhood absence epilepsy. Neurology. (2011) 76:1960–7. 10.1212/WNL.0b013e31821e54de 21646622 PMC3109878

[37] Coenen AM Drinkenburg WH Inoue M van Luijtelaar EL. Genetic models of absence epilepsy, with emphasis on the WAG/Rij strain of rats. Epilepsy Res. (1992) 12:75–86. 10.1016/0920-1211(92)90029-S 1396543

[38] Danober L Deransart C Depaulis A Vergnes M Marescaux C. Pathophysiological mechanisms of genetic absence epilepsy in the rat. Prog Neurobiol. (1998) 55:27–57. 10.1016/S0301-0082(97)00091-9 9602499

[39] Depaulis A David O Charpier S. The genetic absence epilepsy rat from Strasbourg as a model to decipher the neuronal and network mechanisms of generalized idiopathic epilepsies. J Neurosci Methods. (2016) 260:159–74. 10.1016/j.jneumeth.2015.05.022 26068173

[40] PitkäNen A Buckmaster PS Galanopoulou AS Moshé SL. Models of Seizures and Epilepsy. Cambridge: Academic Press (2017).

[41] Depaulis A Charpier S. Pathophysiology of absence epilepsy: insights from genetic models. Neurosci Lett. (2018) 667:53–65. 10.1016/j.neulet.2017.02.035 28216336

[42] Depaulis A Luijtelaar vG. Characteristics of genetic absence seizures in the rat. In: Pitkanen A Schwartzkroin PA Moshe S, editors, Models of Seizure and Epilepsy (London: International: Elsevier Academic Press). (2006). p. 233–48. 10.1016/B978-012088554-1/50020-7

[43] Noebels JL. Single-gene models of epilepsy. Adv Neurol. (1999) 79:227–38. 10514817

[44] Frankel WN. Genetics of complex neurological disease: challenges and opportunities for modeling epilepsy in mice and rats. Trends Genet. (2009) 25:361–7. 10.1016/j.tig.2009.07.001 19665252 PMC2736783

[45] Maheshwari A Noebels JL. Monogenic models of absence epilepsy: windows into the complex balance between inhibition and excitation in thalamocortical microcircuits. Prog Brain Res. (2014) 213:223–52. 10.1016/B978-0-444-63326-2.00012-0 25194492

[46] Meeren HK Pijn JP Van Luijtelaar EL Coenen AM Lopes da Silva FH. Cortical focus drives widespread corticothalamic networks during spontaneous absence seizures in rats. J Neurosci. (2002) 22:1480–95. 10.1523/JNEUROSCI.22-04-01480.2002 11850474 PMC6757554

[47] Manning JP Richards DA Leresche N Crunelli V Bowery NG. Cortical-area specific block of genetically determined absence seizures by ethosuximide. Neuroscience. (2004) 123:5–9. 10.1016/j.neuroscience.2003.09.026 14667436

[48] Polack PO Guillemain I Hu E Deransart C Depaulis A Charpier S. Deep layer somatosensory cortical neurons initiate spike-and-wave discharges in a genetic model of absence seizures. J Neurosci. (2007) 27:6590–9. 10.1523/JNEUROSCI.0753-07.2007 17567820 PMC6672429

[49] Polack PO Mahon S Chavez M Charpier S. Inactivation of the somatosensory cortex prevents paroxysmal oscillations in cortical and related thalamic neurons in a genetic model of absence epilepsy. Cereb Cortex. (2009) 19:2078–91. 10.1093/cercor/bhn237 19276326

[50] Studer F Laghouati E Jarre G David O Pouyatos B Depaulis A. Sensory coding is impaired in rat absence epilepsy. J Physiol. (2019) 597:951–66. 10.1113/JP277297 30548850 PMC6355637

[51] Snead OC. gamma-Hydroxybutyrate model of generalized absence seizures: further characterization and comparison with other absence models. Epilepsia. (1988) 29:361–8. 10.1111/j.1528-1157.1988.tb03732.x 3391142

[52] Snead OC. Pharmacological models of generalized absence seizures in rodents. J Neural Transm Suppl. (1992) 35:7–19. 10.1007/978-3-7091-9206-1_2 1380980

[53] Venzi M Di Giovanni G Crunelli V. A critical evaluation of the gamma-hydroxybutyrate (GHB) model of absence seizures. CNS Neurosci Ther. (2015) 21:123–40. 10.1111/cns.12337 25403866 PMC4335601

[54] Cortez MA Kostopoulos GK Snead OC. Acute and chronic pharmacological models of generalized absence seizures. J Neurosci Methods. (2016) 260:175–84. 10.1016/j.jneumeth.2015.08.034 26343323

[55] Lee S Hwang E Lee M Choi JH. Distinct topographical patterns of spike-wave discharge in transgenic and pharmacologically induced absence seizure models. Exp Neurobiol. (2019) 28:474–84. 10.5607/en.2019.28.4.474 31495076 PMC6751861

[56] van Luijtelaar G Sitnikova E. Global and focal aspects of absence epilepsy: the contribution of genetic models. Neurosci Biobehav Rev. (2006) 30:983–1003. 10.1016/j.neubiorev.2006.03.002 16725200

[57] Lüttjohann A Zhang S de Peijper R van Luijtelaar G. Electrical stimulation of the epileptic focus in absence epileptic WAG/Rij rats: assessment of local and network excitability. Neuroscience. (2011) 188:125–34. 10.1016/j.neuroscience,.2011.04.038 21569824

[58] van Luijtelaar G Behr C Avoli M. Is there such a thing as “generalized” epilepsy? Adv Exp Med Biol. (2014) 813:81–91. 10.1007/978-94-017-8914-1_7 25012369

[59] Paz JT Huguenard JR. Microcircuits and their interactions in epilepsy: is the focus out of focus? Nat Neurosci. (2015) 18:351–9. 10.1038/nn.3950 25710837 PMC4561622

[60] Avoli M. A brief history on the oscillating roles of thalamus and cortex in absence seizures. Epilepsia. (2012) 53:779–89. 10.1111/j.1528-1167.2012.03421.x 22360294 PMC4878899

[61] Lüttjohann A van Luijtelaar G. Dynamics of networks during absence seizure's on- and offset in rodents and man. Front Physiol. (2015) 6:16. 10.3389/fphys.2015.00016 25698972 PMC4318340

[62] Sorokin JM Davidson TJ Frechette E Abramian AM Deisseroth K Huguenard JR. Bidirectional control of generalized epilepsy networks *via* rapid real-time switching of firing mode. Neuron. (2017) 93:194–210. 10.1016/j.neuron.2016.11.026 27989462 PMC5268077

[63] Lüttjohann A Schoffelen JM van Luijtelaar G. Peri-ictal network dynamics of spike-wave discharges: phase and spectral characteristics. Exp Neurol. (2013) 239:235–47. 10.1016/j.expneurol.2012.10.021 23124095

[64] Sysoeva MV Lüttjohann A van Luijtelaar G Sysoev IV. Dynamics of directional coupling underlying spike-wave discharges. Neuroscience. (2016) 314:75–89. 10.1016/j.neuroscience.2015.11.044 26633265

[65] Lüttjohann A Pape HC. Regional specificity of cortico-thalamic coupling strength and directionality during waxing and waning of spike and wave discharges. Sci Rep. (2019) 9:2100. 10.1038/s41598-018-37985-7 30765744 PMC6375974

[66] Zhang W Bruno RM. High-order thalamic inputs to primary somatosensory cortex are stronger and longer lasting than cortical inputs. Elife. (2019) 8:18. 10.7554/eLife.44158.018 30741160 PMC6370338

[67] Bal T von Krosigk M McCormick DA. Role of the ferret perigeniculate nucleus in the generation of synchronized oscillations *in vitro*. J Physiol. (1995) 483:665–85. 10.1113/jphysiol.1995.sp020613 7776250 PMC1157809

[68] Bal T von Krosigk M McCormick DA. Synaptic and membrane mechanisms underlying synchronized oscillations in the ferret lateral geniculate nucleus *in vitro*. J Physiol. (1995) 483:641–63. 10.1113/jphysiol.1995.sp020612 7776249 PMC1157808

[69] McCormick DA Contreras D. On the cellular and network bases of epileptic seizures. Annu Rev Physiol. (2001) 63:815–46. 10.1146/annurev.physiol.63.1.815 11181977

[70] Pinault D. Cellular interactions in the rat somatosensory thalamocortical system during normal and epileptic 5–9 Hz oscillations. J Physiol. (2003) 552:881–905. 10.1113/jphysiol.2003.046573 12923213 PMC2343451

[71] Cain SM Snutch TP. T-type calcium channels in burst-firing, network synchrony, and epilepsy. Biochim Biophys Acta. (2013) 1828:1572–8. 10.1016/j.bbamem.2012.07.028 22885138

[72] Richards DA Lemos T Whitton PS Bowery NG. Extracellular GABA in the ventrolateral thalamus of rats exhibiting spontaneous absence epilepsy: a microdialysis study. J Neurochem. (1995) 65:1674–80. 10.1046/j.1471-4159.1995.65041674.x 7561864

[73] Cope DW Hughes SW Crunelli V. GABAA receptor-mediated tonic inhibition in thalamic neurons. J Neurosci. (2005) 25:11553–63. 10.1523/JNEUROSCI.3362-05.2005 16354913 PMC6726040

[74] Cope DW Di Giovanni G Fyson SJ Orbán G Errington AC Lorincz ML. Enhanced tonic GABAA inhibition in typical absence epilepsy. Nat Med. (2009) 15:1392–8. 10.1038/nm.2058 19966779 PMC2824149

[75] Errington AC Cope DW Crunelli V. Augmentation of tonic GABA(A) inhibition in absence epilepsy: therapeutic value of inverse agonists at extrasynaptic GABA(A) receptors. Adv Pharmacol Sci. (2011) 2011:790590. 10.1155/2011/790590 21912539 PMC3168769

[76] Errington AC Gibson KM Crunelli V Cope DW. Aberrant GABA(A) receptor-mediated inhibition in cortico-thalamic networks of succinic semialdehyde dehydrogenase deficient mice. PLoS ONE. (2011) 6:e19021. 10.1371/journal.pone.0019021 21526163 PMC3079762

[77] Pirttimaki T Parri HR Crunelli V. Astrocytic GABA transporter GAT-1 dysfunction in experimental absence seizures. J Physiol. (2013) 591:823–33. 10.1113/jphysiol.2012.242016 23090943 PMC3591700

[78] Huguenard JR. Perspective: is cortical hyperexcitability the only path to generalized absence epilepsy? Epilepsy Curr. (2020) 20:59S−61S. 10.1177/1535759720959325 33287573 PMC7726732

[79] Bomben VC Aiba I Qian J Mark MD Herlitze S Noebels JL. Isolated P/Q calcium channel deletion in layer VI. Corticothalamic neurons generates absence epilepsy. J Neurosci. (2016) 36:405–18. 10.1523/JNEUROSCI.2555-15.2016 26758833 PMC4710767

[80] Halassa MM Fellin T Haydon PG. The tripartite synapse: roles for gliotransmission in health and disease. Trends Mol Med. (2007) 13:54–63. 10.1016/j.molmed.2006.12.005 17207662

[81] von Bartheld CS Bahney J Herculano-Houzel S. The search for true numbers of neurons and glial cells in the human brain: a review of 150 years of cell counting. J Comp Neurol. (2016) 524:3865–95. 10.1002/cne.24040 27187682 PMC5063692

[82] Allen NJ Lyons DA. Glia as architects of central nervous system formation and function. Science. (2018) 362:181–5. 10.1126/science.aat0473 30309945 PMC6292669

[83] Araque A Parpura V Sanzgiri RP Haydon PG. Tripartite synapses: glia, the unacknowledged partner. Trends Neurosci. (1999) 22:208–15. 10.1016/S0166-2236(98)01349-6 10322493

[84] Bazargani N Attwell D. Astrocyte calcium signaling: the third wave. Nat Neurosci. (2016) 19:182–9. 10.1038/nn.4201 26814587

[85] Poskanzer KE Molofsky AV. Dynamism of an astrocyte *in vivo*: perspectives on identity and function. Annu Rev Physiol. (2018) 80:143–57. 10.1146/annurev-physiol-021317-121125 29166242 PMC5811396

[86] Caudal LC Gobbo D Scheller A Kirchhoff F. The paradox of astroglial Ca^2+^ signals at the interface of excitation and inhibition. Front Cell Neurosci. (2020) 14:609947. 10.3389/fncel.2020.609947 33324169 PMC7726216

[87] Fellin T Haydon PG. Do astrocytes contribute to excitation underlying seizures? Trends Mol Med. (2005) 11:530–3. 10.1016/j.molmed.2005.10.007 16290019

[88] Jabs R Seifert G Steinhäuser C. Astrocytic function and its alteration in the epileptic brain. Epilepsia. (2008) 49(Suppl.2):3–12. 10.1111/j.1528-1167.2008.01488.x 18226167

[89] Wetherington J Serrano G Dingledine R. Astrocytes in the epileptic brain. Neuron. (2008) 58:168–78. 10.1016/j.neuron.2008.04.002 18439402 PMC4124883

[90] Coulter DA Steinhäuser C. Role of astrocytes in epilepsy. Cold Spring Harb Perspect Med. (2015) 5:a022434. 10.1101/cshperspect.a022434 25732035 PMC4355248

[91] Crunelli V Carmignoto G Steinhäuser C. Novel astrocyte targets: new avenues for the therapeutic treatment of epilepsy. Neuroscientist. (2015) 21:62–83. 10.1177/1073858414523320 24609207 PMC4361461

[92] Robel S Sontheimer H. Glia as drivers of abnormal neuronal activity. Nat Neurosci. (2016) 19:28–33. 10.1038/nn.4184 26713746 PMC4966160

[93] Binder DK. Astrocytes: stars of the sacred disease. Epilepsy Curr. (2018) 18:172–9. 10.5698/1535-7597.18.3.172 29950942 PMC6017684

[94] Boison D Steinhäuser C. Epilepsy and astrocyte energy metabolism. Glia. (2018) 66:1235–43. 10.1002/glia.23247 29044647 PMC5903956

[95] Patel DC Tewari BP Chaunsali L Sontheimer H. Neuron-glia interactions in the pathophysiology of epilepsy. Nat Rev Neurosci. (2019) 20:282–97. 10.1038/s41583-019-0126-4 30792501 PMC8558781

[96] Verhoog QP Holtman L Aronica E van Vliet EA. Astrocytes as guardians of neuronal excitability: mechanisms underlying epileptogenesis. Front Neurol. (2020) 11:591690. 10.3389/fneur.2020.591690 33324329 PMC7726323

[97] Buskila Y Bellot-Saez A Morley JW. Generating brain waves, the power of astrocytes. Front Neurosci. (2019) 13:1125. 10.3389/fnins.2019.01125 31680846 PMC6813784

[98] Pacholko AG Wotton CA Bekar LK. Astrocytes-the ultimate effectors of long-range neuromodulatory networks? Front Cell Neurosci. (2020) 14:581075. 10.3389/fncel.2020.581075 33192327 PMC7554522

[99] Wang F Qi X Zhang J Huang JH. Astrocytic modulation of potassium under seizures. Neural Regen Res. (2020) 15:980–7. 10.4103/1673-5374.270295 31823867 PMC7034283

[100] Ma B Buckalew R Du Y Kiyoshi CM Alford CC Wang W. Gap junction coupling confers isopotentiality on astrocyte syncytium. Glia. (2016) 64:214–26. 10.1002/glia.22924 26435164 PMC4595908

[101] Steinhäuser C Grunnet M Carmignoto G. Crucial role of astrocytes in temporal lobe epilepsy. Neuroscience. (2016) 323:157–69. 10.1016/j.neuroscience.2014.12.047 25592426

[102] Bellot-Saez A Kékesi O Morley JW Buskila Y. Astrocytic modulation of neuronal excitability through K. Neurosci Biobehav Rev. (2017) 77:87–97. 10.1016/j.neubiorev.2017.03.002 28279812

[103] Kiyoshi CM Du Y Zhong S Wang W Taylor AT Xiong B. Syncytial isopotentiality: a system-wide electrical feature of astrocytic networks in the brain. Glia. (2018) 66:2756–69. 10.1002/glia.23525 30277621 PMC8818325

[104] Kucheryavykh YV Kucheryavykh LY Nichols CG Maldonado HM Baksi K Reichenbach A. Downregulation of Kir4.1 inward rectifying potassium channel subunits by RNAi impairs potassium transfer and glutamate uptake by cultured cortical astrocytes. Glia. (2007) 55:274–81. 10.1002/glia.20455 17091490

[105] Djukic B Casper KB Philpot BD Chin LS McCarthy KD. Conditional knock-out of Kir4.1 leads to glial membrane depolarization, inhibition of potassium and glutamate uptake, and enhanced short-term synaptic potentiation. J Neurosci. (2007) 27:11354–65. 10.1523/JNEUROSCI.0723-07.2007 17942730 PMC6673037

[106] Haj-Yasein NN Jensen V Vindedal GF Gundersen GA Klungland A Ottersen OP. Evidence that compromised K+ spatial buffering contributes to the epileptogenic effect of mutations in the human Kir4.1 gene (KCNJ10). Glia. (2011) 59:1635–42. 10.1002/glia.21205 21748805

[107] Sicca F Ambrosini E Marchese M Sforna L Servettini I Valvo G. Gain-of-function defects of astrocytic Kir4.1 channels in children with autism spectrum disorders and epilepsy. Sci Rep. (2016) 6:34325. 10.1038/srep34325 27677466 PMC5039625

[108] Scholl UI Choi M Liu T Ramaekers VT Häusler MG Grimmer J. Seizures, sensorineural deafness, ataxia, mental retardation, and electrolyte imbalance (SeSAME syndrome) caused by mutations in KCNJ10. Proc Natl Acad Sci USA. (2009) 106:5842–7. 10.1073/pnas.0901749106 19289823 PMC2656559

[109] Bockenhauer D Feather S Stanescu HC Bandulik S Zdebik AA Reichold M. Epilepsy, ataxia, sensorineural deafness, tubulopathy, and KCNJ10 mutations. N Engl J. Med. (2009) 360:1960–70. 10.1056/NEJMoa0810276 19420365 PMC3398803

[110] Papasavvas CA Parrish RR Trevelyan AJ. Propagating activity in neocortex, mediated by gap junctions and modulated by extracellular potassium. eNeuro. (2020) 7:2020. 10.1523/ENEURO.0387-19.2020 32098762 PMC7096537

[111] Bazzigaluppi P Weisspapir I Stefanovic B Leybaert L Carlen PL. Astrocytic gap junction blockade markedly increases extracellular potassium without causing seizures in the mouse neocortex. Neurobiol Dis. (2017) 101:1–7. 10.1016/j.nbd.2016.12.017 28007587

[112] Mukai T Kinboshi M Nagao Y Shimizu S Ono A Sakagami Y. Antiepileptic drugs elevate astrocytic Kir4.1 expression in the rat limbic region. Front Pharmacol. (2018) 9:845. 10.3389/fphar.2018.00845 30127740 PMC6088221

[113] Walch E Murphy TR Cuvelier N Aldoghmi M Morozova C Donohue J. Astrocyte-selective volume increase in elevated extracellular potassium conditions is mediated by the Na. ASN Neuro. (2020) 12:1759091420967152. 10.1177/1759091420967152 33092407 PMC7586494

[114] Eid T Lee TS Thomas MJ Amiry-Moghaddam M Bjørnsen LP Spencer DD. Loss of perivascular aquaporin 4 may underlie deficient water and K+ homeostasis in the human epileptogenic hippocampus. Proc Natl Acad Sci USA. (2005) 102:1193–8. 10.1073/pnas.0409308102 15657133 PMC545857

[115] Binder DK Steinhäuser C. Functional changes in astroglial cells in epilepsy. Glia. (2006) 54:358–68. 10.1002/glia.20394 16886201

[116] Binder DK Yao X Zador Z Sick TJ Verkman AS Manley GT. Increased seizure duration and slowed potassium kinetics in mice lacking aquaporin-4 water channels. Glia. (2006) 53:631–6. 10.1002/glia.20318 16470808

[117] Strohschein S Hüttmann K Gabriel S Binder DK Heinemann U Steinhäuser C. Impact of aquaporin-4 channels on K+ buffering and gap junction coupling in the hippocampus. Glia. (2011) 59:973–80. 10.1002/glia.21169 21446052

[118] Alvestad S Hammer J Hoddevik EH Skare Ø Sonnewald U Amiry-Moghaddam M. Mislocalization of AQP4 precedes chronic seizures in the kainate model of temporal lobe epilepsy. Epilepsy Res. (2013) 105:30–41. 10.1016/j.eplepsyres.2013.01.006 23357720

[119] Lauderdale K Murphy T Tung T Davila D Binder DK Fiacco TA. Osmotic edema rapidly increases neuronal excitability through activation of NMDA receptor-dependent slow inward currents in juvenile and adult hippocampus. ASN Neuro. (2015) 7:1759091415605115. 10.1177/1759091415605115 26489684 PMC4623564

[120] Szu JI Patel DD Chaturvedi S Lovelace JW Binder DK. Modulation of posttraumatic epileptogenesis in aquaporin-4 knockout mice. Epilepsia. (2020) 61:1503–14. 10.1111/epi.16551 32484924

[121] Murphy TR Davila D Cuvelier N Young LR Lauderdale K Binder DK. Hippocampal and cortical pyramidal neurons swell in parallel with astrocytes during acute hypoosmolar stress. Front Cell Neurosci. (2017) 11:275. 10.3389/fncel.2017.00275 28979186 PMC5611379

[122] Fujiwara H Tenney J Kadis DS Altaye M Spencer C Vannest J. Cortical and subcortical volume differences between Benign Epilepsy with Centrotemporal Spikes and Childhood Absence Epilepsy. Epilepsy Res. (2020) 166:106407. 10.1016/j.eplepsyres.2020.106407 32634725 PMC7494623

[123] Kim EH Shim WH Lee JS Yoon HM Ko TS Yum MS. Altered structural network in newly onset childhood absence epilepsy. J Clin Neurol. (2020) 16:573–80. 10.3988/jcn.2020.16.4.573 33029962 PMC7541981

[124] Orkand RK. Glial-interstitial fluid exchange. Ann N Y Acad Sci. (1986) 481:269–72. 10.1111/j.1749-6632.1986.tb27157.x 3028232

[125] Jin MM Chen Z. Role of gap junctions in epilepsy. Neurosci Bull. (2011) 27:389–406. 10.1007/s12264-011-1944-1 22108816 PMC5560390

[126] Pannasch U Vargová L Reingruber J Ezan P Holcman D Giaume C. Astroglial networks scale synaptic activity and plasticity. Proc Natl Acad Sci USA. (2011) 108:8467–72. 10.1073/pnas.1016650108 21536893 PMC3100942

[127] Pannasch U Rouach N. Emerging role for astroglial networks in information processing: from synapse to behavior. Trends Neurosci. (2013) 36:405–17. 10.1016/j.tins.2013.04.004 23659852

[128] Mylvaganam S Ramani M Krawczyk M Carlen PL. Roles of gap junctions, connexins, and pannexins in epilepsy. Front Physiol. (2014) 5:172. 10.3389/fphys.2014.00172 24847276 PMC4019879

[129] Chever O Dossi E Pannasch U Derangeon M Rouach N. Astroglial networks promote neuronal coordination. Sci Signal. (2016) 9:ra6. 10.1126/scisignal.aad3066 26758214

[130] Lapato AS Tiwari-Woodruff SK. Connexins and pannexins: at the junction of neuro-glial homeostasis and disease. J Neurosci Res. (2018) 96:31–44. 10.1002/jnr.24088 28580666 PMC5749981

[131] Li Q Li QQ Jia JN Liu ZQ Zhou HH Mao XY. Targeting gap junction in epilepsy: perspectives and challenges. Biomed Pharmacother. (2019) 109:57–65. 10.1016/j.biopha.2018.10.068 30396092

[132] Parpura V Verkhratsky A. Homeostatic function of astrocytes: Ca(2+) and Na(+) signalling. Transl Neurosci. (2012) 3:334–44. 10.2478/s13380-012-0040-y 23243501 PMC3520132

[133] Wallraff A Köhling R Heinemann U Theis M Willecke K Steinhäuser C. The impact of astrocytic gap junctional coupling on potassium buffering in the hippocampus. J Neurosci. (2006) 26:5438–47. 10.1523/JNEUROSCI.0037-06.2006 16707796 PMC6675300

[134] Bedner P Dupper A Hüttmann K Müller J Herde MK Dublin P. Astrocyte uncoupling as a cause of human temporal lobe epilepsy. Brain. (2015) 138:1208–22. 10.1093/brain/awv067 25765328 PMC5963418

[135] Manjarrez-Marmolejo J Franco-Pérez J. Gap junction blockers: an overview of their effects on induced seizures in animal models. Curr Neuropharmacol. (2016) 14:759–71. 10.2174/1570159X14666160603115942 27262601 PMC5050393

[136] Chang WP Wu JJ Shyu BC. Thalamic modulation of cingulate seizure activity *via* the regulation of gap junctions in mice thalamocingulate slice. PLoS ONE. (2013) 8:e62952. 10.1371/journal.pone.0062952 23690968 PMC3653920

[137] Gigout S Louvel J Rinaldi D Martin B Pumain R. Thalamocortical relationships and network synchronization in a new genetic model “in mirror” for absence epilepsy. Brain Res. (2013) 1525:39–52. 10.1016/j.brainres.2013.05.044 23743261

[138] Gigout S Louvel J Pumain R. Effects *in vitro* and *in vivo* of a gap junction blocker on epileptiform activities in a genetic model of absence epilepsy. Epilepsy Res. (2006) 69:15–29. 10.1016/j.eplepsyres.2005.12.002 16466906

[139] Proulx E Leshchenko Y Kokarovtseva L Khokhotva V El-Beheiry M Snead OC. Functional contribution of specific brain areas to absence seizures: role of thalamic gap-junctional coupling. Eur J. Neurosci. (2006) 23:489–96. 10.1111/j.1460-9568.2005.04558.x 16420455

[140] Gareri P Condorelli D Belluardo N Citraro R Barresi V Trovato-Salinaro A. Antiabsence effects of carbenoxolone in two genetic animal models of absence epilepsy (WAG/Rij rats and lh/lh mice). Neuropharmacology. (2005) 49:551–63. 10.1016/j.neuropharm.2005.04.012 15936783

[141] Vincze R Péter M Szabó Z Kardos J Héja L Kovács Z. Connexin 43 differentially regulates epileptiform activity in models of convulsive and non-convulsive epilepsies. Front Cell Neurosci. (2019) 13:173. 10.3389/fncel.2019.00173 31133805 PMC6523398

[142] Venance L Piomelli D Glowinski J Giaume C. Inhibition by anandamide of gap junctions and intercellular calcium signalling in striatal astrocytes. Nature. (1995) 376:590–4. 10.1038/376590a0 7637807

[143] Guan X Cravatt BF Ehring GR Hall JE Boger DL Lerner RA. The sleep-inducing lipid oleamide deconvolutes gap junction communication and calcium wave transmission in glial cells. J Cell Biol. (1997) 139:1785–92. 10.1083/jcb.139.7.1785 9412472 PMC2132638

[144] Citraro R Russo E Scicchitano F van Rijn CM Cosco D Avagliano C. Antiepileptic action of N-palmitoylethanolamine through CB1 and PPAR-α receptor activation in a genetic model of absence epilepsy. Neuropharmacology. (2013) 69:115–26. 10.1016/j.neuropharm.2012.11.017 23206503

[145] Chemin J Monteil A Perez-Reyes E Nargeot J Lory P. Direct inhibition of T-type calcium channels by the endogenous cannabinoid anandamide. EMBO J.. (2001) 20:7033–40. 10.1093/emboj/20.24.7033 11742980 PMC125779

[146] Cravatt BF Prospero-Garcia O Siuzdak G Gilula NB Henriksen SJ Boger DL. Chemical characterization of a family of brain lipids that induce sleep. Science. (1995) 268:1506–9. 10.1126/science.7770779 7770779

[147] Lees G Edwards MD Hassoni AA Ganellin CR Galanakis D. Modulation of GABA(A) receptors and inhibitory synaptic currents by the endogenous CNS sleep regulator cis-9,10-octadecenoamide (cOA). Br J. Pharmacol. (1998) 124:873–82. 10.1038/sj.bjp.0701918 9692771 PMC1565467

[148] Medina-Ceja L Salazar-Sánchez JC Ortega-Ibarra J Morales-Villagrán A. Connexins-based hemichannels/channels and their relationship with inflammation, seizures and epilepsy. Int J. Mol Sci. (2019) 20:ijms20235976. 10.3390/ijms20235976 31783599 PMC6929063

[149] Griemsmann S Höft SP Bedner P Zhang J von Staden E Beinhauer A. Characterization of panglial gap junction networks in the thalamus, neocortex, and hippocampus reveals a unique population of glial cells. Cereb Cortex. (2015) 25:3420–33. 10.1093/cercor/bhu157 25037920 PMC4585496

[150] Aquilino MS Whyte-Fagundes P Zoidl G Carlen PL. Pannexin-1 channels in epilepsy. Neurosci Lett. (2019) 695:71–5. 10.1016/j.neulet.2017.09.004 28886985

[151] Scemes E Velíšek L Velíšková J. Astrocyte and neuronal Pannexin1 contribute distinctly to seizures. ASN Neuro. (2019) 11:1759091419833502. 10.1177/1759091419833502 30862176 PMC6415468

[152] Wang J Ma M Locovei S Keane RW Dahl G. Modulation of membrane channel currents by gap junction protein mimetic peptides: size matters. Am J Physiol Cell Physiol. (2007) 293:C1112–9. 10.1152/ajpcell.00097.2007 17652431

[153] Willebrords J Maes M Crespo Yanguas S Vinken M. Inhibitors of connexin and pannexin channels as potential therapeutics. Pharmacol Ther. (2017) 180:144–60. 10.1016/j.pharmthera.2017.07.001 28720428 PMC5802387

[154] Delvaeye T Vandenabeele P Bultynck G Leybaert L Krysko DV. Therapeutic targeting of connexin channels: new views and challenges. Trends Mol Med. (2018) 24:1036–53. 10.1016/j.molmed.2018.10.005 30424929

[155] Giaume C Naus CC Sáez JC Leybaert L. Glial connexins and pannexins in the healthy and diseased brain. Physiol Rev. (2021) 101:93–145. 10.1152/physrev.00043.2018 32326824

[156] Dallérac G Zapata J Rouach N. Versatile control of synaptic circuits by astrocytes: where, when and how? Nat Rev Neurosci. (2018) 19:729–43. 10.1038/s41583-018-0080-6 30401802

[157] Savtchouk I Volterra A. Gliotransmission: beyond black-and-white. J Neurosci. (2018) 38:14–25. 10.1523/JNEUROSCI.0017-17.2017 29298905 PMC6705815

[158] Riquelme J Wellmann M Sotomayor-Zárate R Bonansco C. Gliotransmission: a novel target for the development of antiseizure drugs. Neuroscientist. (2020) 26:293–309. 10.1177/1073858420901474 31976817

[159] Leal A Vieira JP Lopes R Nunes RG Gonçalves SI Lopes da Silva F. Dynamics of epileptic activity in a peculiar case of childhood absence epilepsy and correlation with thalamic levels of GABA. Epilepsy Behav Case Rep. (2016) 5:57–65. 10.1016/j.ebcr.2016.03.004 27144122 PMC4840417

[160] Dikow N Maas B Karch S Granzow M Janssen JW Jauch A. 3p25.3 microdeletion of GABA transporters SLC6A1 and SLC6A11 results in intellectual disability, epilepsy and stereotypic behavior. Am J Med Genet A. (2014) 164A:3061–8. 10.1002/ajmg.a.36761 25256099

[161] Mattison KA Butler KM Inglis GAS Dayan O Boussidan H Bhambhani V. SLC6A1 variants identified in epilepsy patients reduce γ-aminobutyric acid transport. Epilepsia. (2018) 59:e135–41. 10.1111/epi.14531 30132828

[162] Galer PD Ganesan S Lewis-Smith D McKeown SE Pendziwiat M Helbig KL. Semantic similarity analysis reveals robust gene-disease relationships in developmental and epileptic encephalopathies. Am J Hum Genet. (2020) 107:683–97. 10.1016/j.ajhg.2020.08.003 32853554 PMC7536581

[163] Goodspeed K Pérez-Palma E Iqbal S Cooper D Scimemi A Johannesen KM. Current knowledge of SLC6A1-related neurodevelopmental disorders. Brain Commun. (2020) 2:fcaa170. 10.1093/braincomms/fcaa170 33241211 PMC7677605

[164] Johannesen KM Gardella E Linnankivi T Courage C de Saint Martin A Lehesjoki A-E. Defining the phenotypic spectrum of SLC6A1 mutations. Epilepsia. (2018) 59:389–402. 10.1111/epi.13986 29315614 PMC5912688

[165] Jiménez-González C Pirttimaki T Cope DW Parri HR. Non-neuronal, slow GABA signalling in the ventrobasal thalamus targets δ-subunit-containing GABA(A) receptors. Eur J. Neurosci. (2011) 33:1471–82. 10.1111/j.1460-9568.2011.07645.x 21395866 PMC3110310

[166] Herd MB Brown AR Lambert JJ Belelli D. Extrasynaptic GABA(A) receptors couple presynaptic activity to postsynaptic inhibition in the somatosensory thalamus. J Neurosci. (2013) 33:14850–68. 10.1523/JNEUROSCI.1174-13.2013 24027285 PMC6705167

[167] Höft S Griemsmann S Seifert G Steinhäuser C. Heterogeneity in expression of functional ionotropic glutamate and GABA receptors in astrocytes across brain regions: insights from the thalamus. Philos Trans R Soc Lond B Biol Sci. (2014) 369:20130602. 10.1098/rstb.2013.0602 25225096 PMC4173288

[168] Merlo D Mollinari C Inaba Y Cardinale A Rinaldi AM D'Antuono M. Reduced GABAB receptor subunit expression and paired-pulse depression in a genetic model of absence seizures. Neurobiol Dis. (2007) 25:631–41. 10.1016/j.nbd.2006.11.005 17207629

[169] Inaba Y D'Antuono M Bertazzoni G Biagini G Avoli M. Diminished presynaptic GABA(B) receptor function in the neocortex of a genetic model of absence epilepsy. Neurosignals. (2009) 17:121–31. 10.1159/000197864 19176980 PMC4878904

[170] Vergnes M Marescaux C Micheletti G Depaulis A Rumbach L Warter JM. Enhancement of spike and wave discharges by GABAmimetic drugs in rats with spontaneous petit-mal-like epilepsy. Neurosci Lett. (1984) 44:91–4. 10.1016/0304-3940(84)90226-X 6425742

[171] Crunelli V Leresche N. A role for GABAB receptors in excitation and inhibition of thalamocortical cells. Trends Neurosci. (1991) 14:16–21. 10.1016/0166-2236(91)90178-W 1709527

[172] Liu Z Vergnes M Depaulis A Marescaux C. Involvement of intrathalamic GABAB neurotransmission in the control of absence seizures in the rat. Neuroscience. (1992) 48:87–93. 10.1016/0306-4522(92)90340-8 1316571

[173] Marescaux C Vergnes M Bernasconi R. GABAB receptor antagonists: potential new anti-absence drugs. J Neural Transm Suppl. (1992) 35:179–88. 10.1007/978-3-7091-9206-1_12 1324979

[174] Manning JP Richards DA Bowery NG. Pharmacology of absence epilepsy. Trends Pharmacol Sci. (2003) 24:542–9. 10.1016/j.tips.2003.08.006 14559407

[175] Beenhakker MP Huguenard JR. Astrocytes as gatekeepers of GABAB receptor function. J Neurosci. (2010) 30:15262–76. 10.1523/JNEUROSCI.3243-10.2010 21068331 PMC3056552

[176] Bortolato M Frau R Orrù M Fà M Dessì C Puligheddu M. GABAB receptor activation exacerbates spontaneous spike-and-wave discharges in DBA/2J mice. Seizure. (2010) 19:226–31. 10.1016/j.seizure.2010.02.007 20233662

[177] Gould T Chen L Emri Z Pirttimaki T Errington AC Crunelli V. GABA(B) receptor-mediated activation of astrocytes by gamma-hydroxybutyric acid. Philos Trans R Soc Lond B Biol Sci. (2014) 369:20130607. 10.1098/rstb.2013.0607 25225100 PMC4173292

[178] D'Amore V von Randow C Nicoletti F Ngomba RT van Luijtelaar G. Anti-absence activity of mGlu1 and mGlu5 receptor enhancers and their interaction with a GABA reuptake inhibitor: effect of local infusions in the somatosensory cortex and thalamus. Epilepsia. (2015) 56:1141–51. 10.1111/epi.13024 26040777

[179] Ngomba RT Biagioni F Casciato S Willems-van Bree E Battaglia G Bruno V. The preferential mGlu2/3 receptor antagonist, LY341495, reduces the frequency of spike-wave discharges in the WAG/Rij rat model of absence epilepsy. Neuropharmacology. (2005) 49(Suppl.1):89–103. 10.1016/j.neuropharm.2005.05.019 16043198

[180] Ngomba RT Ferraguti F Badura A Citraro R Santolini I Battaglia G. Positive allosteric modulation of metabotropic glutamate 4 (mGlu4) receptors enhances spontaneous and evoked absence seizures. Neuropharmacology. (2008) 54:344–54. 10.1016/j.neuropharm.2007.10.004 18022649

[181] Celli R Santolini I Van Luijtelaar G Ngomba RT Bruno V Nicoletti F. Targeting metabotropic glutamate receptors in the treatment of epilepsy: rationale and current status. Expert Opin Ther Targets. (2019) 23:341–51. 10.1080/14728222.2019.1586885 30801204

[182] Celli R Wall MJ Santolini I Vergassola M Di Menna L Mascio G. Pharmacological activation of mGlu5 receptors with the positive allosteric modulator VU0360172, modulates thalamic GABAergic transmission. Neuropharmacology. (2020) 178:108240. 10.1016/j.neuropharm.2020.108240 32768418

[183] Parri HR Gould TM Crunelli V. Sensory and cortical activation of distinct glial cell subtypes in the somatosensory thalamus of young rats. Eur J. Neurosci. (2010) 32:29–40. 10.1111/j.1460-9568.2010.07281.x 20608967 PMC2909395

[184] Tanaka K Watase K Manabe T Yamada K Watanabe M Takahashi K. Epilepsy and exacerbation of brain injury in mice lacking the glutamate transporter GLT-1. Science. (1997) 276:1699–702. 10.1126/science.276.5319.1699 9180080

[185] Watanabe T Morimoto K Hirao T Suwaki H Watase K Tanaka K. Amygdala-kindled and pentylenetetrazole-induced seizures in glutamate transporter GLAST-deficient mice. Brain Res. (1999) 845:92–6. 10.1016/S0006-8993(99)01945-9 10529447

[186] Coulter DA Eid T. Astrocytic regulation of glutamate homeostasis in epilepsy. Glia. (2012) 60:1215–26. 10.1002/glia.22341 22592998 PMC3375386

[187] Eid T Lee TW Patrylo P Zaveri HP. Astrocytes and glutamine synthetase in epileptogenesis. J Neurosci Res. (2019) 97:1345–62. 10.1002/jnr.24267 30022509 PMC6338538

[188] Dutuit M Touret M Szymocha R Nehlig A Belin MF Didier-Bazès M. Decreased expression of glutamate transporters in genetic absence epilepsy rats before seizure occurrence. J Neurochem. (2002) 80:1029–38. 10.1046/j.0022-3042.2002.00768.x 11953453

[189] Ingram EM Tessler S Bowery NG Emson PC. Glial glutamate transporter mRNAs in the genetically absence epilepsy rat from Strasbourg. Brain Res Mol Brain Res. (2000) 75:96–104. 10.1016/S0169-328X(99)00301-0 10648892

[190] Takano T Kang J Jaiswal JK Simon SM Lin JHC Yu YF. Receptor-mediated glutamate release from volume sensitive channels in astrocytes. Proc Natl Acad Sci USA. (2005) 102:16466–71. 10.1073/pnas.0506382102 16254051 PMC1283436

[191] Héja L Simon Á Szabó Z. Kardos J. Feedback adaptation of synaptic excitability *via* Glu:Na. Neuropharmacology. (2019) 161:107629. 10.1016/j.neuropharm.2019.05.006 31103619

[192] Melø TM Sonnewald U Touret M Nehlig A. Cortical glutamate metabolism is enhanced in a genetic model of absence epilepsy. J Cereb Blood Flow Metab. (2006) 26:1496–506. 10.1038/sj.jcbfm.9600300 16538229

[193] Dutuit M Didier-Bazès M Vergnes M Mutin M Conjard A Akaoka H. Specific alteration in the expression of glial fibrillary acidic protein, glutamate dehydrogenase, and glutamine synthetase in rats with genetic absence epilepsy. Glia. (2000) 32:15–24. 10.1002/1098-1136(200010)32:1<15::AID-GLIA20>3.0.CO;2-# 10975907

[194] Dufour F Nalecz KA Nalecz MJ Nehlig A. Metabolic approach of absence seizures in a genetic model of absence epilepsy, the GAERS: study of the leucine-glutamate cycle. J Neurosci Res. (2001) 66:923–30. 10.1002/jnr.10086 11746420

[195] Bahi-Buisson N El Sabbagh S Soufflet C Escande F Boddaert N Valayannopoulos V. Myoclonic absence epilepsy with photosensitivity and a gain of function mutation in glutamate dehydrogenase. Seizure. (2008) 17:658–64. 10.1016/j.seizure.2008.01.005 18321734

[196] Bazzigaluppi P Ebrahim Amini A Weisspapir I Stefanovic B Carlen PL. Hungry neurons: metabolic insights on seizure dynamics. Int J Mol Sci. (2017) 18:112269. 10.3390/ijms18112269 PMC5713239 29143800

[197] van Rijn CM Gaetani S Santolini I Badura A Gabova A Fu J. WAG/Rij rats show a reduced expression of CB1 receptors in thalamic nuclei and respond to the CB1 receptor agonist, R(+)WIN55,212-2, with a reduced incidence of spike-wave discharges. Epilepsia. (2010) 51:1511–21. 10.1111/j.1528-1167.2009.02510.x 20132294

[198] Citraro R Russo E Ngomba RT Nicoletti F Scicchitano F Whalley BJ. CB1 agonists, locally applied to the cortico-thalamic circuit of rats with genetic absence epilepsy, reduce epileptic manifestations. Epilepsy Res. (2013) 106:74–82. 10.1016/j.eplepsyres.2013.06.004 23860329

[199] Perescis MFJ Flipsen NAR van Luijtelaar G van Rijn CM. Altered SWD stopping mechanism in WAG/Rij rats subchronically treated with the cannabinoid agonist R(+)WIN55,212-2. Epilepsy Behav. (2020) 102:106722. 10.1016/j.yebeh.2019.106722 31855784

[200] Nikolic L Nobili P Shen W Audinat E. Role of astrocyte purinergic signaling in epilepsy. Glia. (2020) 68:1677–91. 10.1002/glia.23747 31705600

[201] Engel T Alves M Sheedy C Henshall DC. ATPergic signalling during seizures and epilepsy. Neuropharmacology. (2016) 104:140–53. 10.1016/j.neuropharm.2015.11.001 26549853

[202] Rassendren F Audinat E. Purinergic signaling in epilepsy. J Neurosci Res. (2016) 94:781–93. 10.1002/jnr.23770 27302739

[203] Weltha L Reemmer J Boison D. The role of adenosine in epilepsy. Brain Res Bull. (2019) 151:46–54. 10.1016/j.brainresbull.2018.11.008 30468847 PMC6527499

[204] Ekonomou A Angelatou F Vergnes M Kostopoulos G. Lower density of A1 adenosine receptors in nucleus reticularis thalami in rats with genetic absence epilepsy. Neuroreport. (1998) 9:2135–40. 10.1097/00001756-199806220-00042 9674608

[205] D'Alimonte I D'Auro M Citraro R Biagioni F Jiang S Nargi E. Altered distribution and function of A2A adenosine receptors in the brain of WAG/Rij rats with genetic absence epilepsy, before and after appearance of the disease. Eur J Neurosci. (2009) 30:1023–35. 10.1111/j.1460-9568.2009.06897.x 19723291

[206] Lakatos RK Dobolyi Á Todorov MI Kékesi KA Juhász G Aleksza M. Guanosine may increase absence epileptic activity by means of A2A adenosine receptors in Wistar Albino Glaxo Rijswijk rats. Brain Res Bull. (2016) 124:172–81. 10.1016/j.brainresbull.2016.05.001 27154620

[207] Ilbay G Sahin D Karson A Ates N. Effects of adenosine administration on spike-wave discharge frequency in genetically epileptic rats. Clin Exp Pharmacol Physiol. (2001) 28:643–6. 10.1046/j.1440-1681.2001.03499.x 11473530

[208] Germé K Faure JB Koning E Nehlig A. Effect of caffeine and adenosine receptor ligands on the expression of spike-and-wave discharges in Genetic Absence Epilepsy Rats from Strasbourg (GAERS). Epilepsy Res. (2015) 110:105–14. 10.1016/j.eplepsyres.2014.11.022 25616462

[209] Kovács Z D'Agostino DP Dobolyi A Ari C. Adenosine A1 receptor antagonism abolished the anti-seizure effects of exogenous ketone supplementation in wistar albino glaxo rijswijk rats. Front Mol Neurosci. (2017) 10:235. 10.3389/fnmol.2017.00235 28790891 PMC5524776

[210] Muhle H Steinich I von Spiczak S Franke A Weber Y Lerche H. A duplication in 1q21.3 in a family with early onset and childhood absence epilepsy. Epilepsia. (2010) 51:2453–6. 10.1111/j.1528-1167.2010.02712.x 21204805

[211] Chen CP Lin SP Chen M Su YN Chern SR Wang TY. Mosaic supernumerary r(1)(p13.2q23.3) in a 10-year-old girl with epilepsy facial asymmetry psychomotor retardation kyphoscoliosis dermatofibrosarcoma and multiple exostoses. Genet Couns. (2011) 22:273–80. 22029168

[212] Sun QQ Huguenard JR Prince DA. Neuropeptide Y. receptors differentially modulate G-protein-activated inwardly rectifying K+ channels and high-voltage-activated Ca^2^+ channels in rat thalamic neurons. J Physiol. (2001) 531:67–79. 10.1111/j.1469-7793.2001.0067j.x 11179392 PMC2278450

[213] Elms J Powell KL van Raay L Dedeurwaerdere S O'Brien TJ Morris MJ. Long-term valproate treatment increases brain neuropeptide Y. expression and decreases seizure expression in a genetic rat model of absence epilepsy. PLoS ONE. (2013) 8:e73505. 10.1371/journal.pone.0073505 24039965 PMC3767750

[214] Stroud LM O'Brien TJ Jupp B Wallengren C Morris MJ. Neuropeptide Y. suppresses absence seizures in a genetic rat model. Brain Res. (2005) 1033:151–6. 10.1016/j.brainres.2004.11.022 15694919

[215] Morris MJ Gannan E Stroud LM Beck-Sickinger AG O'Brien TJ. Neuropeptide Y. suppresses absence seizures in a genetic rat model primarily through effects on Y. receptors. Eur J. Neurosci. (2007) 25:1136–43. 10.1111/j.1460-9568.2007.05348.x 17331209

[216] van Raay L Jovanovska V Morris MJ O'Brien TJ. Focal administration of neuropeptide Y. into the S2 somatosensory cortex maximally suppresses absence seizures in a genetic rat model. Epilepsia. (2012) 53:477–84. 10.1111/j.1528-1167.2011.03370.x 22220638

[217] Woldbye DP Nanobashvili A Sørensen AT Husum H Bolwig TG Sørensen G. Differential suppression of seizures *via* Y2 and Y5 neuropeptide Y. receptors. Neurobiol Dis. (2005) 20:760–72. 10.1016/j.nbd.2005.05.010 15979311

[218] Lin EJ Young D Baer K Herzog H During MJ. Differential actions of NPY on seizure modulation *via* Y1 and Y2 receptors: evidence from receptor knockout mice. Epilepsia. (2006) 47:773–80. 10.1111/j.1528-1167.2006.00500.x 16650144

[219] Powell KL Fitzgerald X Shallue C Jovanovska V Klugmann M Von Jonquieres G. Gene therapy mediated seizure suppression in Genetic Generalised Epilepsy: Neuropeptide Y. overexpression in a rat model. Neurobiol Dis. (2018) 113:23–32. 10.1016/j.nbd.2018.01.016 29414380

[220] Ramamoorthy P Whim MD. Trafficking and fusion of neuropeptide Y-containing dense-core granules in astrocytes. J Neurosci. (2008) 28:13815–27. 10.1523/JNEUROSCI.5361-07.2008 19091972 PMC2635891

[221] Schwarz Y Zhao N Kirchhoff F Bruns D. Astrocytes control synaptic strength by two distinct v-SNARE-dependent release pathways. Nat Neurosci. (2017) 20:1529–39. 10.1038/nn.4647 28945220

[222] Gimpl G Kirchhoff F Lang RE Kettenmann H. Identification of neuropeptide Y. receptors in cultured astrocytes from neonatal rat brain. J Neurosci Res. (1993) 34:198–205. 10.1002/jnr.490340207 8450563

[223] Barnea A Aguila-Mansilla N Bigio EH Worby C Roberts J. Evidence for regulated expression of neuropeptide Y. gene by rat and human cultured astrocytes. Regul Pept. (1998) 75–76:293–300. 10.1016/S0167-0115(98)00081-0 9802422

[224] Carmignoto G Haydon PG. Astrocyte calcium signaling and epilepsy. Glia. (2012) 60:1227–33. 10.1002/glia.22318 22389222 PMC4532388

[225] Shigetomi E Saito K Sano F Koizumi S. Aberrant Calcium signals in reactive astrocytes: a key process in neurological disorders. Int J. Mol Sci. (2019) 20:40996. 10.3390/ijms20040996 30823575 PMC6413203

[226] Parri HR Gould TM Crunelli V. Spontaneous astrocytic Ca^2^+ oscillations *in situ* drive NMDAR-mediated neuronal excitation. Nat Neurosci. (2001) 4:803–12. 10.1038/90507 11477426

[227] Angulo MC Kozlov AS Charpak S Audinat E. Glutamate released from glial cells synchronizes neuronal activity in the hippocampus. J Neurosci. (2004) 24:6920–7. 10.1523/JNEUROSCI.0473-04.2004 15295027 PMC6729611

[228] Fellin T Pascual O Gobbo S Pozzan T Haydon PG Carmignoto G. Neuronal synchrony mediated by astrocytic glutamate through activation of extrasynaptic NMDA receptors. Neuron. (2004) 43:729–43. 10.1016/j.neuron.2004.08.011 15339653

[229] Tian GF Azmi H Takano T Xu QW Peng WG Lin J. An astrocytic basis of epilepsy. Nat Med. (2005) 11:973–81. 10.1038/nm1277 16116433 PMC1850946

[230] Gómez-Gonzalo M Losi G Chiavegato A Zonta M Cammarota M Brondi M. An excitatory loop with astrocytes contributes to drive neurons to seizure threshold. PLoS Biol. (2010) 8:e1000352. 10.1371/journal.pbio.1000352 20405049 PMC2854117

[231] Koizumi S. Synchronization of Ca^2^+ oscillations: involvement of ATP release in astrocytes. FEBS J. (2010) 277:286–92. 10.1111/j.1742-4658.2009.07438.x 19895581

[232] Sasaki T Ishikawa T Abe R Nakayama R Asada A Matsuki N. Astrocyte calcium signalling orchestrates neuronal synchronization in organotypic hippocampal slices. J Physiol. (2014) 592:2771–83. 10.1113/jphysiol.2014.272864 24710057 PMC4221819

[233] Álvarez-Ferradas C Morales JC Wellmann M Nualart F Roncagliolo M Fuenzalida M. Enhanced astroglial Ca^2^+ signaling increases excitatory synaptic strength in the epileptic brain. Glia. (2015) 63:1507–21. 10.1002/glia.22817 25980474

[234] Wellmann M Álvarez-Ferradas C Maturana CJ Sáez JC Bonansco C. Astroglial Ca^2+^-dependent hyperexcitability requires P2Y_1_purinergic receptors and pannexin-1 channel activation in a chronic model of epilepsy. Front Cell Neurosci. (2018) 12:446. 10.3389/fncel.2018.00446 30542266 PMC6277884

[235] Heuser K Nome CG Pettersen KH Åbjørsbråten KS Jensen V Tang W. Ca^2^+ Signals in astrocytes facilitate spread of epileptiform activity. Cereb Cortex. (2018) 28:4036–48. 10.1093/cercor/bhy196 30169757 PMC6188565

[236] Seidel JL Escartin C Ayata C Bonvento G Shuttleworth CW. Multifaceted roles for astrocytes in spreading depolarization: a target for limiting spreading depolarization in acute brain injury? Glia. (2016) 64:5–20. 10.1002/glia.22824 26301517 PMC4715804

[237] Parri HR Crunelli V. Pacemaker calcium oscillations in thalamic astrocytes *in situ*. Neuroreport. (2001) 12:3897–900. 10.1097/00001756-200112210-00008 11742206

[238] Yu X Taylor AM. W. Nagai J Golshani P Evans CJ Coppola G. Reducing astrocyte calcium signaling *in vivo* alters striatal microcircuits and causes repetitive behavior. Neuron. (2018) 99:1170–87.e1179. 10.1016/j.neuron.2018.08.015 30174118 PMC6450394

[239] Pirttimaki TM Hall SD Parri HR. Sustained neuronal activity generated by glial plasticity. J Neurosci. (2011) 31:7637–47. 10.1523/JNEUROSCI.5783-10.2011 21613477 PMC3118423

[240] Pirttimaki TM Parri HR. Glutamatergic input-output properties of thalamic astrocytes. Neuroscience. (2012) 205:18–28. 10.1016/j.neuroscience.2011.12.049 22233780 PMC3314995

[241] Copeland CS Wall TM Sims RE Neale SA Nisenbaum E Parri HR. Astrocytes modulate thalamic sensory processing *via* mGlu2 receptor activation. Neuropharmacology. (2017) 121:100–10. 10.1016/j.neuropharm.2017.04.019 28416443 PMC5480778

[242] Christian CA Huguenard JR. Astrocytes potentiate GABAergic transmission in the thalamic reticular nucleus *via* endozepine signaling. Proc Natl Acad Sci USA. (2013) 110:20278–83. 10.1073/pnas.1318031110 24262146 PMC3864346

[243] Pirttimaki TM Sims RE Saunders G Antonio SA Codadu NK Parri HR. Astrocyte-mediated neuronal synchronization properties revealed by false gliotransmitter release. J Neurosci. (2017) 37:9859–70. 10.1523/JNEUROSCI.2761-16.2017 28899919 PMC5637115

[244] Kékesi O Ioja E Szabó Z Kardos J Héja L. Recurrent seizure-like events are associated with coupled astroglial synchronization. Front Cell Neurosci. (2015) 9:215. 10.3389/fncel.2015.00215 26150770 PMC4471369

[245] Ujita S Sasaki T Asada A Funayama K Gao M Mikoshiba K. cAMP-dependent calcium oscillations of astrocytes: an implication for pathology. Cereb Cortex. (2017) 27:1602–14. 10.1093/cercor/bhv310 26803165

[246] Sofroniew MV. Astrogliosis. Cold Spring Harb Perspect Biol. (2014) 7:a020420. 10.1101/cshperspect.a020420 25380660 PMC4315924

[247] Dossi E Vasile F Rouach N. Human astrocytes in the diseased brain. Brain Res Bull. (2018) 136:139–56. 10.1016/j.brainresbull.2017.02.001 28212850 PMC5766741

[248] Escartin C Guillemaud O Carrillo-de Sauvage MA. Questions and (some) answers on reactive astrocytes. Glia. (2019) 67:2221–47. 10.1002/glia.23687 31429127

[249] Robel S Buckingham SC Boni JL Campbell SL Danbolt NC Riedemann T. Reactive astrogliosis causes the development of spontaneous seizures. J Neurosci. (2015) 35:3330–45. 10.1523/JNEUROSCI.1574-14.2015 25716834 PMC4339349

[250] Robel S. Astroglial scarring and seizures: a cell biological perspective on epilepsy. Neuroscientist. (2017) 23:152–68. 10.1177/1073858416645498 27118807

[251] Noè F Cattalini A Vila Verde D Alessi C Colciaghi F Figini M. Epileptiform activity contralateral to unilateral hippocampal sclerosis does not cause the expression of brain damage markers. Epilepsia. (2019) 60:1184–99. 10.1111/epi.15611 31111475

[252] Çavdar S Kuvvet Y Sur-Erdem I Özgür M Onat F. Relationships between astrocytes and absence epilepsy in rat: an experimental study. Neurosci Lett. (2019) 712:134518. 10.1016/j.neulet.2019.134518 31560994

[253] Sitnikova E Kulikova S Birioukova L Raevsky VV. Cellular neuropathology of absence epilepsy in the neocortex: a population of glial cells rather than neurons is impaired in genetic rat model. Acta Neurobiol Exp. (2011) 71:263–8. 21731079 10.55782/ane-2011-1846

[254] Oberheim NA Tian GF Han X Peng W Takano T Ransom B. Loss of astrocytic domain organization in the epileptic brain. J Neurosci. (2008) 28:3264–76. 10.1523/JNEUROSCI.4980-07.2008 18367594 PMC6670598

[255] van Vliet EA Aronica E Gorter JA. Blood-brain barrier dysfunction, seizures and epilepsy. Semin Cell Dev Biol. (2015) 38:26–34. 10.1016/j.semcdb.2014.10.003 25444846

[256] Rana A Musto AE. The role of inflammation in the development of epilepsy. J Neuroinflammation. (2018) 15:144. 10.1186/s12974-018-1192-7 29764485 PMC5952578

[257] Vezzani A Balosso S Ravizza T. Neuroinflammatory pathways as treatment targets and biomarkers in epilepsy. Nat Rev Neurol. (2019) 15:459–72. 10.1038/s41582-019-0217-x 31263255

[258] Terrone G Balosso S Pauletti A Ravizza T Vezzani A. Inflammation and reactive oxygen species as disease modifiers in epilepsy. Neuropharmacology. (2020) 167:107742. 10.1016/j.neuropharm.2019.107742 31421074

[259] Akin D Ravizza T Maroso M Carcak N Eryigit T Vanzulli I. IL-1β is induced in reactive astrocytes in the somatosensory cortex of rats with genetic absence epilepsy at the onset of spike-and-wave discharges, and contributes to their occurrence. Neurobiol Dis. (2011) 44:259–69. 10.1016/j.nbd.2011.05.015 21645619

[260] van Luijtelaar G Lyashenko S Vastyanov R Verbeek G Oleinik A van Rijn C. Cytokines and Absence Seizures in a Genetic Rat Model. Neurophysiology. (2012) 43:478–86. 10.1007/s11062-012-9252-6

[261] Fine SM Angel RA Perry SW Epstein LG Rothstein JD Dewhurst S. Tumor necrosis factor alpha inhibits glutamate uptake by primary human astrocytes. Implications for pathogenesis of HIV-1 dementia. J Biol Chem. (1996) 271:15303–6. 10.1074/jbc.271.26.15303 8663435

[262] Stellwagen D Beattie EC Seo JY Malenka RC. Differential regulation of AMPA receptor and GABA receptor trafficking by tumor necrosis factor-alpha. J Neurosci. (2005) 25:3219–28. 10.1523/JNEUROSCI.4486-04.2005 15788779 PMC6725093

[263] Kovács Z Kékesi KA Szilágyi N Abrahám I Székács D Király N. Facilitation of spike-wave discharge activity by lipopolysaccharides in Wistar Albino Glaxo/Rijswijk rats. Neuroscience. (2006) 140:731–42. 10.1016/j.neuroscience.2006.02.023 16616432

[264] Kovács Z Czurkó A Kékesi KA Juhász G. Intracerebroventricularly administered lipopolysaccharide enhances spike-wave discharges in freely moving WAG/Rij rats. Brain Res Bull. (2011) 85:410–6. 10.1016/j.brainresbull.2011.05.003 21619914

[265] Russo E Citraro R Donato G Camastra C Iuliano R Cuzzocrea S. mTOR inhibition modulates epileptogenesis, seizures and depressive behavior in a genetic rat model of absence epilepsy. Neuropharmacology. (2013) 69:25–36. 10.1016/j.neuropharm.2012.09.019 23092918

[266] Russo E Andreozzi F Iuliano R Dattilo V Procopio T Fiume G. Early molecular and behavioral response to lipopolysaccharide in the WAG/Rij rat model of absence epilepsy and depressive-like behavior, involves interplay between AMPK, AKT/mTOR pathways and neuroinflammatory cytokine release. Brain Behav Immun. (2014) 42:157–68. 10.1016/j.bbi.2014.06.016 24998197

[267] Kovács Z Dobolyi A Juhász G Kékesi KA. Lipopolysaccharide induced increase in seizure activity in two animal models of absence epilepsy WAG/Rij and GAERS rats and Long Evans rats. Brain Res Bull. (2014) 104:7–18. 10.1016/j.brainresbull.2014.03.003 24704320

[268] Leo A Nesci V Tallarico M Amodio N Gallo Cantafio EM De Sarro G. IL-6 receptor blockade by tocilizumab has anti-absence and anti-epileptogenic effects in the WAG/Rij rat model of absence epilepsy. Neurotherapeutics. (2020) 17:2004–14. 10.1007/s13311-020-00893-8 32681356 PMC7851197

[269] Billiau AD Witters P Ceulemans B Kasran A Wouters C Lagae L. Intravenous immunoglobulins in refractory childhood-onset epilepsy: effects on seizure frequency, EEG activity, and cerebrospinal fluid cytokine profile. Epilepsia. (2007) 48:1739–49. 10.1111/j.1528-1167.2007.01134.x 17521345

[270] Steinborn B Zarowski M Winczewska-Wiktor A Wójcicka M Młodzikowska-Albrecht J Losy J. Concentration of Il-1β, Il-2, Il-6, TNFα in the blood serum in children with generalized epilepsy treated by valproate. Pharmacol Rep. (2014) 66:972–5. 10.1016/j.pharep.2014.06.005 25443723

[271] Nir Y Massimini M Boly M Tononi G. Sleep and consciousness. In: Cavanna A Nani A Blumenfeld H Laureys S, editors. Neuroimaging of Consciousness. Berlin; Heidelberg: Springer (2013). p. 133–82. 10.1007/978-3-642-37580-4_9

[272] Beenhakker MP Huguenard JR. Neurons that fire together also conspire together: is normal sleep circuitry hijacked to generate epilepsy? Neuron. (2009) 62:612–32. 10.1016/j.neuron.2009.05.015 19524522 PMC2748990

[273] Contreras D Steriade M. Cellular basis of EEG slow rhythms: a study of dynamic corticothalamic relationships. J Neurosci. (1995) 15:604–22. 10.1523/JNEUROSCI.15-01-00604.1995 7823167 PMC6578315

[274] Steriade M Contreras D Curró Dossi R Nuñez A. The slow (<1 Hz) oscillation in reticular thalamic and thalamocortical neurons: scenario of sleep rhythm generation in interacting thalamic and neocortical networks. J Neurosci. (1993) 13:3284–99. 10.1523/JNEUROSCI.13-08-03284.1993 8340808 PMC6576531

[275] Halász P Terzano MG Parrino L. Spike-wave discharge and the microstructure of sleep-wake continuum in idiopathic generalised epilepsy. Neurophysiol Clin. (2002) 32:38–53. 10.1016/S0987-7053(01)00290-8 11915485

[276] Steriade M Contreras D. Relations between cortical and thalamic cellular events during transition from sleep patterns to paroxysmal activity. J Neurosci. (1995) 15:623–42. 10.1523/JNEUROSCI.15-01-00623.1995 7823168 PMC6578306

[277] McCormick DA Bal T. Sleep and arousal: thalamocortical mechanisms. Annu Rev Neurosci. (1997) 20:185–215. 10.1146/annurev.neuro.20.1.185 9056712

[278] Steriade M. Neuronal Substrates of Sleep and Epilepsy. Cambridge: Cambridge University Press. (2003). p. 322–48.

[279] Steriade M Timofeev I. Neuronal plasticity in thalamocortical networks during sleep and waking oscillations. Neuron. (2003) 37:563–76. 10.1016/S0896-6273(03)00065-5 12597855

[280] Crunelli V David F Lorincz ML Hughes SW. The thalamocortical network as a single slow wave-generating unit. Curr Opin Neurobiol. (2015) 31:72–80. 10.1016/j.conb.2014.09.001 25233254

[281] Krishnan GP Chauvette S Shamie I Soltani S Timofeev I Cash SS. Cellular and neurochemical basis of sleep stages in the thalamocortical network. Elife. (2016) 5:16. 10.7554/eLife.18607.016 27849520 PMC5111887

[282] Halász P Szucs A. Sleep and epilepsy link by plasticity. Front Neurol. (2020) 11:911. 10.3389/fneur.2020.00911 32982931 PMC7491282

[283] Jin B Aung T Geng Y Wang S. Epilepsy and its interaction with sleep and circadian rhythm. Front Neurol. (2020) 11:327. 10.3389/fneur.2020.00327 32457690 PMC7225332

[284] Smyk MK van Luijtelaar G. Circadian rhythms and epilepsy: a suitable case for absence epilepsy. Front Neurol. (2020) 11:245. 10.3389/fneur.2020.00245 32411068 PMC7198737

[285] Xu C Yu J Ruan Y Wang Y Chen Z. Decoding circadian rhythm and epileptic activities: clues from animal studies. Front Neurol. (2020) 11:751. 10.3389/fneur.2020.00751 32793110 PMC7393483

[286] Gloor P. Generalized cortico-reticular epilepsies. Some considerations on the pathophysiology of generalized bilaterally synchronous spike and wave discharge. Epilepsia. (1968) 9:249–63. 10.1111/j.1528-1157.1968.tb04624.x 4975028

[287] Kostopoulos GK. Spike-and-wave discharges of absence seizures as a transformation of sleep spindles: the continuing development of a hypothesis. Clin Neurophysiol. (2000) 111(Suppl.2):S27–38. 10.1016/S1388-2457(00)00399-0 10996552

[288] Kellaway P Frost JD Crawley JW. Time modulation of spike-and-wave activity in generalized epilepsy. Ann Neurol. (1980) 8:491–500. 10.1002/ana.410080506 7192070

[289] Nobili L Baglietto MG Beelke M De Carli F Veneselli E Ferrillo F. Temporal relationship of generalized epileptiform discharges to spindle frequency activity in childhood absence epilepsy. Clin Neurophysiol. (2001) 112:1912–6. 10.1016/S1388-2457(01)00624-1 11595151

[290] Kostopoulos G Gloor P Pellegrini A Gotman J. A study of the transition from spindles to spike and wave discharge in feline generalized penicillin epilepsy: microphysiological features. Exp Neurol. (1981) 73:55–77. 10.1016/0014-4886(81)90045-5 7250289

[291] Fan D Liao F Wang Q. The pacemaker role of thalamic reticular nucleus in controlling spike-wave discharges and spindles. Chaos. (2017) 27:073103. 10.1063/1.4991869 28764392

[292] Halász P Bódizs R Ujma PP Fabó D Szucs A. Strong relationship between NREM sleep, epilepsy and plastic functions - a conceptual review on the neurophysiology background. Epilepsy Res. (2019) 150:95–105. 10.1016/j.eplepsyres.2018.11.008 30712997

[293] Sitnikova E Grubov V Hramov AE. Slow-wave activity preceding the onset of 10–15-Hz sleep spindles and 5-9-Hz oscillations in electroencephalograms in rats with and without absence seizures. J Sleep Res. (2020) 29:e12927. 10.1111/jsr.12927 31578791

[294] Meeren HK Veening JG Möderscheim TA Coenen AM van Luijtelaar G. Thalamic lesions in a genetic rat model of absence epilepsy: dissociation between spike-wave discharges and sleep spindles. Exp Neurol. (2009) 217:25–37. 10.1016/j.expneurol.2009.01.009 19416679

[295] Leresche N Lambert RC Errington AC Crunelli V. From sleep spindles of natural sleep to spike and wave discharges of typical absence seizures: is the hypothesis still valid? Pflugers Arch. (2012) 463:201–12. 10.1007/s00424-011-1009-3 21861061 PMC3256322

[296] Kozák G Földi T Berényi A. Spike-and-wave discharges are not pathological sleep spindles, network-level aspects of age-dependent absence seizure development in rats. eNeuro. (2020) 7:201+9. 10.1523/ENEURO.0253-19.2019 31862790 PMC6944477

[297] Steriade M Nunez A Amzica F. Intracellular analysis of relations between the slow (<1 Hz) neocortical oscillation and other sleep rhythms of the electroencephalogram. J Neurosci. (1993) 13:3266. 10.1523/JNEUROSCI.13-08-03266.1993 8340807 PMC6576520

[298] Steriade M Nuñez A Amzica F. A novel slow (<1 Hz) oscillation of neocortical neurons *in vivo*: depolarizing and hyperpolarizing components. J Neurosci. (1993) 13:3252–65. 10.1523/JNEUROSCI.13-08-03252.1993 8340806 PMC6576541

[299] Crunelli V Hughes SW. The slow (<1 Hz) rhythm of non-REM sleep: a dialogue between three cardinal oscillators. Nat Neurosci. (2010) 13:9–17. 10.1038/nn.2445 19966841 PMC2980822

[300] Neske GT. The slow oscillation in cortical and thalamic networks: mechanisms and functions. Front Neural Circuits. (2015) 9:88. 10.3389/fncir.2015.00088 26834569 PMC4712264

[301] Fiáth R Kerekes BP Wittner L Tóth K Beregszászi P Horváth D. Laminar analysis of the slow wave activity in the somatosensory cortex of anesthetized rats. Eur J. Neurosci. (2016) 44:1935–51. 10.1111/ejn.13274 27177594

[302] Lannes B Micheletti G Vergnes M Marescaux C Depaulis A Warter JM. Relationship between spike-wave discharges and vigilance levels in rats with spontaneous petit mal-like epilepsy. Neurosci Lett. (1988) 94:187–91. 10.1016/0304-3940(88)90293-5 3149401

[303] Terzano MG Parrino L Anelli S Halasz P. Modulation of generalized spike-and-wave discharges during sleep by cyclic alternating pattern. Epilepsia. (1989) 30:772–81. 10.1111/j.1528-1157.1989.tb05337.x 2591344

[304] Coenen AM Drinkenburg WH Peeters BW Vossen JM van Luijtelaar EL. Absence epilepsy and the level of vigilance in rats of the WAG/Rij strain. Neurosci Biobehav Rev. (1991) 15:259–63. 10.1016/S0149-7634(05)80005-3 1906586

[305] Drinkenburg WH Coenen AM Vossen JM Van Luijtelaar EL. Spike-wave discharges and sleep-wake states in rats with absence epilepsy. Epilepsy Res. (1991) 9:218–24. 10.1016/0920-1211(91)90055-K 1743184

[306] Tucker DM Waters AC Holmes MD. Transition from cortical slow oscillations of sleep to spike-wave seizures. Clin Neurophysiol. (2009) 120:2055–62. 10.1016/j.clinph.2009.07.047 19879188

[307] Koutroumanidis M Tsiptsios D Kokkinos V Kostopoulos GK. Focal and generalized EEG paroxysms in childhood absence epilepsy: topographic associations and distinctive behaviors during the first cycle of non-REM sleep. Epilepsia. (2012) 53:840–9. 10.1111/j.1528-1167.2012.03424.x 22360352

[308] Smyk MK Sysoev IV Sysoeva MV van Luijtelaar G Drinkenburg WH. Can absence seizures be predicted by vigilance states? advanced analysis of sleep-wake states and spike-wave discharges' occurrence in rats. Epilepsy Behav. (2019) 96:200–9. 10.1016/j.yebeh.2019.04.012 31153123

[309] Durazzo TS Spencer SS Duckrow RB Novotny EJ Spencer DD Zaveri HP. Temporal distributions of seizure occurrence from various epileptogenic regions. Neurology. (2008) 70:1265–71. 10.1212/01.wnl.0000308938.84918.3f 18391158

[310] Gurkas E Serdaroglu A Hirfanoglu T Kartal A Yilmaz U Bilir E. Sleep-wake distribution and circadian patterns of epileptic seizures in children. Eur J Paediatr Neurol. (2016) 20:549–54. 10.1016/j.ejpn.2016.04.004 27140809

[311] Loddenkemper T Vendrame M Zarowski M Gregas M Alexopoulos AV Wyllie E. Circadian patterns of pediatric seizures. Neurology. (2011) 76:145–53. 10.1212/WNL.0b013e318206ca46 21220719

[312] Zarowski M Loddenkemper T Vendrame M Alexopoulos AV Wyllie E Kothare SV. Circadian distribution and sleep/wake patterns of generalized seizures in children. Epilepsia. (2011) 52:1076–83. 10.1111/j.1528-1167.2011.03023.x 21426332

[313] Halász P. Sleep, arousal and electroclinical manifestations of generalized epilepsy with spike wave pattern. Epilepsy Res Suppl. (1991) 2:43–8. 1662047

[314] Minecan D Natarajan A Marzec M Malow B. Relationship of epileptic seizures to sleep stage and sleep depth. Sleep. (2002) 25:899–904. 10.1093/sleep/25.8.56 12489898

[315] Seneviratne U Lai A Cook M D'Souza W Boston RC. “Sleep Surge”: the impact of sleep onset and offset on epileptiform discharges in idiopathic generalized epilepsies. Clin Neurophysiol. (2020) 131:1044–50. 10.1016/j.clinph.2020.01.021 32199394

[316] Van Luijtelaar EL Coenen AM. Circadian rhythmicity in absence epilepsy in rats. Epilepsy Res. (1988) 2:331–6. 10.1016/0920-1211(88)90042-3 3143564

[317] Smyk MK Coenen AM Lewandowski MH van Luijtelaar G. Endogenous rhythm of absence epilepsy: relationship with general motor activity and sleep-wake states. Epilepsy Res. (2011) 93:120–7. 10.1016/j.eplepsyres.2010.11.003 21146957

[318] Smyk MK van Luijtelaar G Huysmans H Drinkenburg WH. Spike-wave discharges and sleep-wake states during circadian desynchronization: no effects of agomelatine upon re-entrainment. Neuroscience. (2019) 408:327–38. 10.1016/j.neuroscience.2019.03.062 30978380

[319] Smyk MK Coenen A Lewandowski MH van Luijtelaar G. Internal desynchronization facilitates seizures. Epilepsia. (2012) 53:1511–8. 10.1111/j.1528-1167.2012.03577.x 22780432

[320] Kovács Z Slézia A Bali ZK Kovács P Dobolyi A Szikra T. Uridine modulates neuronal activity and inhibits spike-wave discharges of absence epileptic Long Evans and Wistar Albino Glaxo/Rijswijk rats. Brain Res Bull. (2013) 97:16–23. 10.1016/j.brainresbull.2013.05.009 23707857

[321] Honda K Komoda Y Nishida S Nagasaki H Higashi A Uchizono K. Uridine as an active component of sleep-promoting substance: its effects on nocturnal sleep in rats. Neurosci Res. (1984) 1:243–52. 10.1016/S0168-0102(84)80003-6 6549543

[322] Halász P Filakovszky J Vargha A Bagdy G. Effect of sleep deprivation on spike-wave discharges in idiopathic generalised epilepsy: a 4 x 24 h continuous long term EEG monitoring study. Epilepsy Res. (2002) 51:123–32. 10.1016/S0920-1211(02)00123-7 12350388

[323] Giorgi FS Perini D Maestri M Guida M Pizzanelli C Caserta A. Usefulness of a simple sleep-deprived EEG protocol for epilepsy diagnosis in *de novo* subjects. Clin Neurophysiol. (2013) 124:2101–7. 10.1016/j.clinph.2013.04.342 23790524

[324] Rosenow F Klein KM Hamer HM. Non-invasive EEG evaluation in epilepsy diagnosis. Expert Rev Neurother. (2015) 15:425–44. 10.1586/14737175.2015.1025382 25779862

[325] Renzel R Baumann CR Poryazova R. EEG after sleep deprivation is a sensitive tool in the first diagnosis of idiopathic generalized but not focal epilepsy. Clin Neurophysiol. (2016) 127:209–13. 10.1016/j.clinph.2015.06.012 26118491

[326] Van Luijtelaar EL Van der Werf SJ Vossen JM Coenen AM. Arousal, performance and absence seizures in rats. Electroencephalogr Clin Neurophysiol. (1991) 79:430–4. 10.1016/0013-4694(91)90208-L 1718716

[327] Osterhagen L Breteler M van Luijtelaar G. Does arousal interfere with operant conditioning of spike-wave discharges in genetic epileptic rats? Epilepsy Res. (2010) 90:75–82. 10.1016/j.eplepsyres.2010.03.010 20388587

[328] Sudbrack-Oliveira P Lima Najar L Foldvary-Schaefer N da Mota Gomes M. Sleep architecture in adults with epilepsy: a systematic review. Sleep Med. (2019) 53:22–7. 10.1016/j.sleep.2018.09.004 30388678

[329] van Luijtelaar G Bikbaev A. Midfrequency cortico-thalamic oscillations and the sleep cycle: genetic, time of day and age effects. Epilepsy Res. (2007) 73:259–65. 10.1016/j.eplepsyres.2006.11.002 17156975

[330] Yi PL Chen YJ Lin CT Chang FC. Occurrence of epilepsy at different zeitgeber times alters sleep homeostasis differently in rats. Sleep. (2012) 35:1651–65. 10.5665/sleep.2238 23204608 PMC3490358

[331] Krueger JM Fang J Taishi P Chen Z Kushikata T Gardi J. Sleep. A physiologic role for IL-1 beta and TNF-alpha. Ann N Y Acad Sci. (1998) 856:148–59. 10.1111/j.1749-6632.1998.tb08323.x 9917875

[332] Györffy B Kovács Z Gulyássy P Simor A Völgyi K Orbán G. Brain protein expression changes in WAG/Rij rats, a genetic rat model of absence epilepsy after peripheral lipopolysaccharide treatment. Brain Behav Immun. (2014) 35:86–95. 10.1016/j.bbi.2013.09.001 24021561

[333] Lipton JO Boyle LM Yuan ED Hochstrasser KJ Chifamba FF Nathan A. Aberrant proteostasis of BMAL1 underlies circadian abnormalities in a paradigmatic mTOR-opathy. Cell Rep. (2017) 20:868–80. 10.1016/j.celrep.2017.07.008 28746872 PMC5603761

[334] Cooper JM Halter KA Prosser RA. Circadian rhythm and sleep-wake systems share the dynamic extracellular synaptic milieu. Neurobiol Sleep Circadian Rhythms. (2018) 5:15–36. 10.1016/j.nbscr.2018.04.001 31236509 PMC6584685

[335] Re CJ Batterman AI Gerstner JR Buono RJ Ferraro TN. The molecular genetic interaction between circadian rhythms and susceptibility to seizures and epilepsy. Front Neurol. (2020) 11:520. 10.3389/fneur.2020.00520 32714261 PMC7344275

[336] Lananna BV Nadarajah CJ Izumo M Cedeño MR Xiong DD Dimitry J. Cell-autonomous regulation of astrocyte activation by the circadian clock protein BMAL1. Cell Rep. (2018) 25:1–9.e5. 10.1016/j.celrep.2018.09.015 30282019 PMC6221830

[337] Brancaccio M Edwards MD Patton AP Smyllie NJ Chesham JE Maywood ES. Cell-autonomous clock of astrocytes drives circadian behavior in mammals. Science. (2019) 363:187–92. 10.1126/science.aat4104 30630934 PMC6440650

[338] Brancaccio M Patton AP Chesham JE Maywood ES Hastings MH. Astrocytes control circadian timekeeping in the suprachiasmatic nucleus *via* glutamatergic signaling. Neuron. (2017) 93:1420–35.e1425. 10.1016/j.neuron.2017.02.030 28285822 PMC5376383

[339] Hablitz LM Gunesch AN Cravetchi O Moldavan M Allen CN. Cannabinoid signaling recruits astrocytes to modulate presynaptic function in the suprachiasmatic nucleus. eNeuro. (2020) 7:2020. 10.1523/ENEURO.0081-19.2020 31964686 PMC7029187

[340] Chrobok L Palus K Jeczmien-Lazur JS Chrzanowska A Kepczynski M Lewandowski MH. Disinhibition of the intergeniculate leaflet network in the WAG/Rij rat model of absence epilepsy. Exp Neurol. (2017) 289:103–16. 10.1016/j.expneurol.2016.12.014 28041911

[341] Haydon PG. Astrocytes and the modulation of sleep. Curr Opin Neurobiol. (2017) 44:28–33. 10.1016/j.conb.2017.02.008 28284099 PMC5511068

[342] Garofalo S Picard K Limatola C Nadjar A Pascual O Tremblay M. Role of Glia in the regulation of sleep in health and disease. Compr Physiol. (2020) 10:687–712. 10.1002/cphy.c190022 32163207

[343] Xie L Kang H Xu Q Chen MJ Liao Y Thiyagarajan M. Sleep drives metabolite clearance from the adult brain. Science. (2013) 342:373–7. 10.1126/science.1241224 24136970 PMC3880190

[344] Ding F O'Donnell J Xu Q Kang N Goldman N Nedergaard M. Changes in the composition of brain interstitial ions control the sleep-wake cycle. Science. (2016) 352:550–5. 10.1126/science.aad4821 27126038 PMC5441687

[345] Sherpa AD Xiao F Joseph N Aoki C Hrabetova S. Activation of β-adrenergic receptors in rat visual cortex expands astrocytic processes and reduces extracellular space volume. Synapse. (2016) 70:307–16. 10.1002/syn.21908 27085090 PMC4909535

[346] Baskey G Singh A Sharma R Mallick BN. REM sleep deprivation-induced noradrenaline stimulates neuronal and inhibits glial Na-K ATPase in rat brain: *in vivo* and *in vitro* studies. Neurochem Int. (2009) 54:65–71. 10.1016/j.neuint.2008.10.006 19013490

[347] Vyazovskiy VV Olcese U Lazimy YM Faraguna U Esser SK Williams JC. Cortical firing and sleep homeostasis. Neuron. (2009) 63:865–78. 10.1016/j.neuron.2009.08.024 19778514 PMC2819325

[348] Watson BO Levenstein D Greene JP Gelinas JN Buzsáki G. Network homeostasis and state dynamics of neocortical sleep. Neuron. (2016) 90:839–52. 10.1016/j.neuron.2016.03.036 27133462 PMC4873379

[349] Cucchiara F Frumento P Banfi T Sesso G Di Galante M D'Ascanio P. Electrophysiological features of sleep in children with Kir4.1 channel mutations and Autism-Epilepsy phenotype: a preliminary study. Sleep. (2020) 43:zsz255. 10.1093/sleep/zsz255 31722434 PMC7157183

[350] Bellesi M de Vivo L Tononi G Cirelli C. Effects of sleep and wake on astrocytes: clues from molecular and ultrastructural studies. BMC Biol. (2015) 13:66. 10.1186/s12915-015-0176-7 26303010 PMC4548305

[351] DiNuzzo M Nedergaard M. Brain energetics during the sleep-wake cycle. Curr Opin Neurobiol. (2017) 47:65–72. 10.1016/j.conb.2017.09.010 29024871 PMC5732842

[352] Benveniste H Lee H Volkow ND. The glymphatic pathway: waste removal from the CNS *via* cerebrospinal fluid transport. Neuroscientist. (2017) 23:454–65. 10.1177/1073858417691030 28466758 PMC5547012

[353] Hablitz LM Vinitsky HS Sun Q Stæger FF Sigurdsson B Mortensen KN. Increased glymphatic influx is correlated with high EEG delta power and low heart rate in mice under anesthesia. Sci Adv. (2019) 5:eaav5447. 10.1126/sciadv.aav5447 30820460 PMC6392807

[354] Hablitz LM Plá V Giannetto M Vinitsky HS Stæger FF Metcalfe T. Circadian control of brain glymphatic and lymphatic fluid flow. Nat Commun. (2020) 11:4411. 10.1038/s41467-020-18115-2 32879313 PMC7468152

[355] Ulv Larsen SM Landolt HP Berger W Nedergaard M Knudsen GM Holst SC. Haplotype of the astrocytic water channel AQP4 is associated with slow wave energy regulation in human NREM sleep. PLoS Biol. (2020) 18:e3000623. 10.1371/journal.pbio.3000623 32369477 PMC7199924

[356] Petit JM Magistretti PJ. Regulation of neuron-astrocyte metabolic coupling across the sleep-wake cycle. Neuroscience. (2016) 323:135–56. 10.1016/j.neuroscience.2015.12.007 26704637

[357] Clasadonte J Scemes E Wang Z Boison D Haydon PG. Connexin 43-mediated astroglial metabolic networks contribute to the regulation of the sleep-wake cycle. Neuron. (2017) 95:1365–80.e1365. 10.1016/j.neuron.2017.08.022 28867552 PMC5617118

[358] Petit JM Gyger J Burlet-Godinot S Fiumelli H Martin JL Magistretti PJ. Genes involved in the astrocyte-neuron lactate shuttle (ANLS) are specifically regulated in cortical astrocytes following sleep deprivation in mice. Sleep. (2013) 36:1445–58. 10.5665/sleep.3034 24082304 PMC3773194

[359] Sada N Lee S Katsu T Otsuki T Inoue T. Epilepsy treatment. Targeting LDH enzymes with a stiripentol analog to treat epilepsy. Science. (2015) 347:1362–7. 10.1126/science.aaa1299 25792327

[360] Fisher JL. The anti-convulsant stiripentol acts directly on the GABA(A) receptor as a positive allosteric modulator. Neuropharmacology. (2009) 56:190–7. 10.1016/j.neuropharm.2008.06.004 18585399 PMC2665930

[361] Halassa MM Florian C Fellin T Munoz JR Lee S.-Y Abel T. Astrocytic modulation of sleep homeostasis and cognitive consequences of sleep loss. Neuron. (2009) 61:213–9. 10.1016/j.neuron.2008.11.024 19186164 PMC2673052

[362] Ursin R Bjorvatn B. Sleep-wake and eeg effects following adenosine a1 agonism and antagonism: similarities and interactions with sleep-wake and eeg effects following a serotonin reuptake inhibitor in rats. Sleep Res Online. (1998) 1:119–27. 11382868

[363] Thakkar MM Winston S McCarley RW. A1 receptor and adenosinergic homeostatic regulation of sleep-wakefulness: effects of antisense to the A1 receptor in the cholinergic basal forebrain. J Neurosci. (2003) 23:4278–87. 10.1523/JNEUROSCI.23-10-04278.2003 12764116 PMC2002519

[364] Blutstein T Haydon PG. The Importance of astrocyte-derived purines in the modulation of sleep. Glia. (2013) 61:129–39. 10.1002/glia.22422 23027687 PMC3527671

[365] Nadjar A Blutstein T Aubert A Laye S Haydon PG. Astrocyte-derived adenosine modulates increased sleep pressure during inflammatory response. Glia. (2013) 61:724–31. 10.1002/glia.22465 23378051

[366] Zhou X Oishi Y Cherasse Y Korkutata M Fujii S Lee CY. Extracellular adenosine and slow-wave sleep are increased after ablation of nucleus accumbens core astrocytes and neurons in mice. Neurochem Int. (2019) 124:256–63. 10.1016/j.neuint.2019.01.020 30690114

[367] Fellin T Halassa MM Terunuma M Succol F Takano H Frank M. Endogenous non-neuronal modulators of synaptic transmission control cortical slow oscillations *in vivo*. Proc Natl Acad Sci USA. (2009) 106:15037–42. 10.1073/pnas.0906419106 19706442 PMC2736412

[368] Poskanzer KE Yuste R. Astrocytes regulate cortical state switching *in vivo*. Proc Natl Acad Sci USA. (2016) 113:E2675–84. 10.1073/pnas.1520759113 27122314 PMC4868485

[369] Szabó Z Héja L Szalay G Kékesi O Füredi A Szebényi K. Extensive astrocyte synchronization advances neuronal coupling in slow wave activity *in vivo*. Sci Rep. (2017) 7:6018. 10.1038/s41598-017-06073-7 28729692 PMC5519671

[370] Wang M He Y Sejnowski TJ Yu X. Brain-state dependent astrocytic Ca^2+^ signals are coupled to both positive and negative BOLD-fMRI signals. Proc Natl Acad Sci USA. (2018) 115:E1647–56. 10.1073/pnas.1711692115 29382752 PMC5816146

[371] Brockett AT Kane GA Monari PK Briones BA Vigneron PA Barber GA. Evidence supporting a role for astrocytes in the regulation of cognitive flexibility and neuronal oscillations through the Ca^2^+ binding protein S100β. PLoS ONE. (2018) 13:e0195726. 10.1371/journal.pone.0195726 29664924 PMC5903631

[372] Foley J Blutstein T Lee S Erneux C Halassa MM Haydon P. Astrocytic IP_3_/Ca^2+^ signaling modulates theta rhythm and REM sleep. Front Neural Circuits. (2017) 11:3. 10.3389/fncir.2017.00003 28167901 PMC5253379

[373] Bojarskaite L Bjørnstad DM Pettersen KH Cunen C Hermansen GH Åbjørsbråten KS. Astrocytic Ca^2+^signaling is reduced during sleep and is involved in the regulation of slow wave sleep. Nat Commun. (2020) 11:3240. 10.1038/s41467-020-17062-2 32632168 PMC7338360

[374] Takahashi K Kayama Y Lin JS Sakai K. Locus coeruleus neuronal activity during the sleep-waking cycle in mice. Neuroscience. (2010) 169:1115–26. 10.1016/j.neuroscience.2010.06.009 20542093

[375] Wang Y Burghardt TP Worrell GA Wang HL. The frequency-dependent effect of electrical fields on the mobility of intracellular vesicles in astrocytes. Biochem Biophys Res Commun. (2020) 22:111286. 10.1101/2020.05.22.111286 33280815 PMC8215681

[376] El Helou J Bélanger-Nelson E Freyburger M Dorsaz S Curie T La Spada F. Neuroligin-1 links neuronal activity to sleep-wake regulation. Proc Natl Acad Sci USA. (2013) 110:9974–9. 10.1073/pnas.1221381110 23716671 PMC3683757

[377] Reissner C Runkel F Missler M. Neurexins. Genome Biol. (2013) 14:213. 10.1186/gb-2013-14-9-213 24083347 PMC4056431

[378] Rudenko G. Dynamic control of synaptic adhesion and organizing molecules in synaptic plasticity. Neural Plast. (2017) 2017:6526151. 10.1155/2017/6526151 28255461 PMC5307005

[379] Massart R Freyburger M Suderman M Paquet J El Helou J Belanger-Nelson E. The genome-wide landscape of DNA methylation and hydroxymethylation in response to sleep deprivation impacts on synaptic plasticity genes. Transl Psychiatry. (2014) 4:e347. 10.1038/tp.2013.120 24448209 PMC3905230

[380] Singh SK Stogsdill JA Pulimood NS Dingsdale H Kim YH Pilaz LJ. Astrocytes assemble thalamocortical synapses by bridging NRX1α and NL1 *via* Hevin. Cell. (2016) 164:183–96. 10.1016/j.cell.2015.11.034 26771491 PMC4715262

[381] Cao F Liu JJ Zhou S Cortez MA Snead OC Han J. Neuroligin. 2 regulates absence seizures and behavioral arrests through GABAergic transmission within the thalamocortical circuitry. Nat Commun. (2020) 11:3744. 10.1038/s41467-020-17560-3 32719346 PMC7385104

[382] Matsuki T Takasu M Hirose Y Murakoshi N Sinton CM Motoike T. GABAA receptor-mediated input change on orexin neurons following sleep deprivation in mice. Neuroscience. (2015) 284:217–24. 10.1016/j.neuroscience.2014.09.063 25286384

[383] Liu JJ Grace KP Horner RL Cortez MA Shao Y Jia Z. Neuroligin. 3 R451C mutation alters electroencephalography spectral activity in an animal model of autism spectrum disorders. Mol Brain. (2017) 10:10. 10.1186/s13041-017-0290-2 28385162 PMC5384041

[384] Iasevoli F Tomasetti C de Bartolomeis A. Scaffolding proteins of the post-synaptic density contribute to synaptic plasticity by regulating receptor localization and distribution: relevance for neuropsychiatric diseases. Neurochem Res. (2013) 38:1–22. 10.1007/s11064-012-0886-y 22991141

[385] O'Connor EC Bariselli S Bellone C. Synaptic basis of social dysfunction: a focus on postsynaptic proteins linking group-I mGluRs with AMPARs and NMDARs. Eur J. Neurosci. (2014) 39:1114–29. 10.1111/ejn.12510 24712991

[386] Lesca G Rudolf G Labalme A Hirsch E Arzimanoglou A Genton P. Epileptic encephalopathies of the Landau-Kleffner and continuous spike and waves during slow-wave sleep types: genomic dissection makes the link with autism. Epilepsia. (2012) 53:1526–38. 10.1111/j.1528-1167.2012.03559.x 22738016

[387] Holder JL Quach MM. The spectrum of epilepsy and electroencephalographic abnormalities due to SHANK3 loss-of-function mutations. Epilepsia. (2016) 57:1651–9. 10.1111/epi.13506 27554343 PMC5547884

[388] Imeri L Opp MR. How (and why) the immune system makes us sleep. Nat Rev Neurosci. (2009) 10:199–210. 10.1038/nrn2576 19209176 PMC2839418

[389] Krueger JM Clinton JM Winters BD Zielinski MR Taishi P Jewett KA. Involvement of cytokines in slow wave sleep. Prog Brain Res. (2011) 193:39–47. 10.1016/B978-0-444-53839-0.00003-X 21854954 PMC3645329

[390] Irwin MR Opp MR. Sleep health: reciprocal regulation of sleep and innate immunity. Neuropsychopharmacology. (2017) 42:129–55. 10.1038/npp.2016.148 27510422 PMC5143488

[391] Del Gallo F Opp MR Imeri L. The reciprocal link between sleep and immune responses. Arch Ital Biol. (2014) 152:93–102. 10.12871/000298292014234 25828681

[392] Fang J Wang Y Krueger JM. Mice lacking the TNF55 kDa receptor fail to sleep more after TNFalpha treatment. J Neurosci. (1997) 17:5949–55. 10.1523/JNEUROSCI.17-15-05949.1997 9221791 PMC6573218

[393] Fang J Wang Y Krueger JM. Effects of interleukin-1 beta on sleep are mediated by the type I. receptor. Am J. Physiol. (1998) 274:R655–60. 10.1152/ajpregu.1998.274.3.R655 9530230

[394] Krueger JM Taishi P De A Davis CJ Winters BD Clinton J. ATP and the purine type 2 X7 receptor affect sleep. J Appl Physiol. (2010) 109:1318–27. 10.1152/japplphysiol.00586.2010 20829501 PMC2980381

[395] Kovalzon VM Moiseenko LS Ambaryan AV Kurtenbach S Shestopalov VI Panchin YV. Sleep-wakefulness cycle and behavior in pannexin1 knockout mice. Behav Brain Res. (2017) 318:24–7. 10.1016/j.bbr.2016.10.015 27769744

[396] Yoshida H Peterfi Z García-García F Kirkpatrick R Yasuda T Krueger JM. State-specific asymmetries in EEG slow wave activity induced by local application of TNFalpha. Brain Res. (2004) 1009:129–36. 10.1016/j.brainres.2004.02.055 15120590

[397] Churchill L Yasuda K Yasuda T Blindheim KA Falter M Garcia-Garcia F. Unilateral cortical application of tumor necrosis factor alpha induces asymmetry in Fos- and interleukin-1beta-immunoreactive cells within the corticothalamic projection. Brain Res. (2005) 1055:15–24. 10.1016/j.brainres.2005.06.052 16098952

[398] Murphy S Simmons ML Agullo L Garcia A Feinstein DL Galea E. Synthesis of nitric oxide in CNS glial cells. Trends Neurosci. (1993) 16:323–8. 10.1016/0166-2236(93)90109-Y 7691008

[399] Wong ML Rettori V al-Shekhlee A Bongiorno PB Canteros G McCann SM. Inducible nitric oxide synthase gene expression in the brain during systemic inflammation. Nat Med. (1996) 2:581–4. 10.1038/nm0596-581 8616720

[400] Banach M Piskorska B Czuczwar SJ Borowicz KK. Nitric oxide, epileptic seizures, and action of antiepileptic drugs. CNS Neurol Disord Drug Targets. (2011) 10:808–19. 10.2174/187152711798072347 21999730

[401] Sharma S Puttachary S Thippeswamy T. Glial source of nitric oxide in epileptogenesis: a target for disease modification in epilepsy. J Neurosci Res. (2019) 97:1363–77. 10.1002/jnr.24205 29230865 PMC6035106

[402] Brown RE Basheer R McKenna JT Strecker RE McCarley RW. Control of sleep and wakefulness. Physiol Rev. (2012) 92:1087–7. 10.1152/physrev.00032.2011 22811426 PMC3621793

[403] Cespuglio R Amrouni D Meiller A Buguet A Gautier-Sauvigné S. Nitric oxide in the regulation of the sleep-wake states. Sleep Med Rev. (2012) 16:265–79. 10.1016/j.smrv.2012.01.006 22406306

[404] Kalinchuk AV Stenberg D Rosenberg PA Porkka-Heiskanen T. Inducible and neuronal nitric oxide synthases (NOS) have complementary roles in recovery sleep induction. Eur J. Neurosci. (2006) 24:1443–56. 10.1111/j.1460-9568.2006.05019.x 16987226

